# Evaluation of an open-face 8-channel transmit 64-channel receive 7T head coil for neuroimaging

**DOI:** 10.3389/fnins.2026.1811488

**Published:** 2026-06-19

**Authors:** Natasha E. Fullerton, Belinda Ding, Sydney N. Williams, Sarah Allwood-Spiers, Paul McElhinney, Divya Baskaran, Rosie Woodward, Tracey Hopkins, Evonne McLennan, Steven Winata, Janhavi Ghosalkar, Hao Gao, Graeme A. Keith, Eva McEwan, Nicolas Boulant, Paul Donnelly, Laura Preston, Katy Macdonald, Ian M. McLaughlin, Russell Overend, David A. Porter, Shajan Gunamony

**Affiliations:** 1 Imaging Centre of Excellence, School of Psychology and Neuroscience, University of Glasgow, Glasgow, United Kingdom; 2 Department of Neuroradiology, Institute of Neurological Sciences, Queen Elizabeth University Hospital, National Health Service (NHS) Greater Glasgow and Clyde, Glasgow, United Kingdom; 3 Siemens Healthcare Ltd., Camberley, United Kingdom; 4 Medical Physics, University Hospitals Birmingham National Health Service (NHS) Foundation Trust, Birmingham, United Kingdom; 5 Medical Image Analysis and Biometry Lab, Universidad Rey Juan Carlos, Madrid, Spain; 6 Department of Clinical Physics and Bioengineering, National Health Service (NHS) Greater Glasgow and Clyde, Glasgow, United Kingdom; 7 School of Medicine, Dentistry and Nursing, University of Glasgow, Glasgow, United Kingdom; 8 MR CoilTech Limited, Glasgow, United Kingdom; 9 Clinical Research Imaging Facility, Research and Innovation, National Health Service (NHS) Greater Glasgow and Clyde, Glasgow, United Kingdom; 10 School of Cardiovascular and Metabolic Health, University of Glasgow, Glasgow, United Kingdom; 11 School of Mathematics and Statistics, University of Glasgow, Glasgow, United Kingdom; 12 Université Paris-Saclay, Commissariat à l'énergie atomique et aux énergies alternatives (CEA), NeuroSpin, Gif Sur Yvette, France; 13 Department of Radiology, Queen Elizabeth University Hospital, National Health Service (NHS) Greater Glasgow and Clyde, Glasgow, United Kingdom

**Keywords:** 64 channel receiver array, 7T, clinical MRI, neuroimaging, parallel-transmit, radiofrequency head coil, ultra-high field MRI, validation study

## Abstract

**Introduction:**

Ultra-high field (UHF) 7 tesla (7T) MRI offers unique diagnostic opportunities for clinical neuroimaging. However, broader clinical implementation remains limited because the reduced radiofrequency (RF) wavelength at 7T causes RF transmit field (${\text{B}}_{1}^{+}$) inhomogeneity, resulting in spatial variation in image signal and tissue contrast. These effects are particularly pronounced in the skull base, temporal lobes, and posterior fossa, regions frequently implicated in neurological disease. RF parallel transmission (pTx) techniques can improve ${\text{B}}_{1}^{+}$ homogeneity through optimization of multiple transmit fields, while parallel imaging with multiple receive elements enables accelerated acquisitions. To fully exploit these advantages at UHF, RF coils combining high-performance transmit and high-density receive arrays are required.

**Methods:**

An open-face eight-channel transmit 64-channel receive (8Tx64Rx) 7T head coil was developed with electrical and mechanical ergonomic features designed to improve usability and imaging homogeneity. The coil was evaluated in an in vivo validation study assessing ease of use, participant comfort, diagnostic value, and image quality using questionnaires and structured scoring sheets. Imaging performance of the 8Tx64Rx coil was compared with that of a commercially available regulatory approved 7T head coil featuring single transmit (sTx) and a 32-channel receive array. Diagnostic image quality acquired using circularly polarized (CP) mode and pTx mode was additionally assessed and compared.

**Results:**

The 8Tx64Rx coil demonstrated image quality that was equivalent or superior to the commercial 7T head coil across evaluated measures. In particular, improved signal homogeneity was achieved within the posterior fossa and temporal lobes. The coil also supported practical clinical use through favorable ergonomic performance and user comfort during scanning. Diagnostic image quality was enhanced when using pTx approaches compared with conventional transmit configurations.

**Discussion:**

The developed 8Tx64Rx 7T head coil demonstrates the potential to advance clinical neuroimaging at UHF by improving ${\text{B}}_{1}^{+}$ homogeneity in anatomically challenging brain regions while maintaining usability and diagnostic performance. These findings support the integration of combined high-density receive arrays and pTx technology to overcome key limitations of 7T MRI and facilitate broader clinical adoption.

## 1 Introduction

Ultra-high field (UHF) 7T MRI offers promising advantages over clinical neuroimaging at 3T (Ladd et al., 2018; Polimeni and Uludag, 2018; Keith et al., 2024). The increased signal-to-noise ratio (SNR) at higher magnetic field strength may be translated into higher spatial resolution, faster acquisition, and improved contrast-to-noise ratios (Schick, 2005; Pohmann et al., 2015). These gains can be clinically significant, as the ability to resolve fine anatomical detail at 7T can enhance the detection of subtle abnormalities, such as cortical plaques in multiple sclerosis (MS), cortical dysplasias and other migrational abnormalities in epilepsy, often undetected at lower field strengths (Bruschi et al., 2020; Fullerton et al., 2024; Harrison et al., 2024; Opheim et al., 2021; Zampeli et al., 2026). In addition, altered relaxation properties and stronger susceptibility-related dephasing at higher field strength support advanced contrast mechanisms, improving techniques such as susceptibility-weighted imaging (SWI) (Deistung et al., 2008; Balchandani and Naidich, 2015), time-of-flight MR angiography (TOF-MRA) (Balchandani and Naidich, 2015; Lakhani et al., 2022), and functional MRI (fMRI) techniques (Viessmann and Polimeni, 2021), which rely on T_2_-weighted gradient echo sequences This opens up 7T MRI to clinical vascular imaging, with improved detection of microvascular pathology and haemosiderin deposition and staining. TOF-MRA at 7T makes it possible to image the intracranial vessels of the Circle of Willis (CoW) at high resolution with an exquisite diagnostic quality, hitherto only achieved with more invasive neuro-interventional techniques, such as digital-subtraction angiography (DSA) (Harteveld et al., 2015; Cosottini et al., 2024). The increased susceptibility dephasing at UHF also leads to improved detection of mineralization and quantification of mineral deposition using quantitative susceptibility mapping (QSM), a potential imaging biomarker for pathologies such as MS and motor neuron disease (MND) (Aker et al., 2021; Costagli et al., 2016; Spincemaille et al., 2019; de Vries et al., 2025). UHF imaging additionally benefits from increased spectral dispersion, leading to detection of metabolites not easily identified at lower field strength, such as separation of glutamate and glutamine peaks, and detection of disease-specific metabolites, such as the oncometabolite 2-hydroxyglutarate (2HG) (Tkáč et al., 2001; Ugurbil et al., 2003). This enables identification of novel imaging biomarkers in various pathologies, in particular glial brain tumors, but also pathologies such as MND, and non-invasive biochemical sampling of tissues and lesions (Ganji et al., 2016; Cheong et al., 2017; Hangel et al., 2019).

With continued technical development and the first 7T systems being cleared for clinical use by the FDA and CE-marked in Europe in 2017, UHF MRI has begun to transition from primarily a research modality to a tool with clinical potential. However, clinical implementation of 7T MR creates different challenges from a research environment. For example, it requires robust operation across diverse patient populations, a broad range of imaging protocols, and consistent diagnostic quality throughout the brain rather than within selected regions. However, despite regulatory approvals facilitating clinical use, the clinical application of 7T MRI remains limited. This is largely due to difficulties arising from the reducing radiofrequency (RF) wavelength in tissues at higher field strength, which causes ${\text{B}}_{1}^{+}$ transmit field inhomogeneity, spatial variation in flip angle, signal dropout, and reduced contrast uniformity (Schick, 2005; Kraff and Quick, 2017). This spatial variation in image signal and tissue contrast affects especially the skull base, temporal lobes, and posterior fossa, including brainstem and cerebellum. This signal in-homogeneity strongly affects clinical neuroimaging, which relies on high quality, diagnostic sequences of the whole brain. Neurological diseases, such as brain tumors and MS, affect the whole brain including the posterior fossa and temporal lobes, while some disorders preferentially involve the temporal lobes, such as temporal lobe epilepsy and may be amenable to surgical treatment if an epileptogenic focus is identified. As the potential for tissue heating due to RF power deposition increases with the square of the static field strength, specific absorption rate (SAR) restrictions represent an additional constraint.

One solution for addressing ${\text{B}}_{1}^{+}$ field inhomogeneity at 7T is the use of high permittivity dielectric pads, which are placed in areas of the brain to improve areas of low RF sensitivity (Teeuwisse et al., 2011). Dielectric pads have been used for a variety of neurological applications, improving 7T MRI sequences that are particularly sensitivity to ${\text{B}}_{1}^{+}$ inhomogeneity, such as diffusion-weighted imaging (DWI) (Vu et al., 2015) and arterial spin labeling (ASL) (Kashyap et al., 2021). Nevertheless, dielectric pads have limitations to their use, namely that their electromagnetic configuration is fixed at the point of fabrication (non-specific to the subject) (Maurya and Schmidt, 2024; Maurya et al., 2025) and there is a need for further, elaborate analysis and simulation of further potential increases in SAR (Brink et al., 2023).

Parallel RF transmission (pTx) has emerged as an effective means of mitigating the UHF ${\text{B}}_{1}^{+}$ field and SAR challenges by enabling spatial control of the transmit field to homogenize RF excitation and potentially provide more efficient RF energy distribution (Kraff and Quick, 2017; Williams et al., 2023). In the context of UHF, pTx methods improve image uniformity and recover diminishing areas of signal drop-out, such as in the temporal lobes. In pTx, a dedicated RF coil is driven with multiple transmit channels, most often to achieve increased homogeneity of ${\text{B}}_{1}^{+}$ (Hoult, 2000; Ibrahim et al., 2000). Unlike the use of dielectric pads, pTx allows subject and geometry-specific adjustment of ${\text{B}}_{1}^{+}$. Two common forms of pTx exist: static pTx, where per-channel RF complex weights are varied but remain constant during the RF pulse, or dynamic pTx, where RF and gradient waveforms vary over the duration of the pulse, enabling spatiotemporal control of excitation. In addition to RF excitation homogeneity, pTx can also accelerate spatially selective RF pulses to improve the localized imaging of targeted tissue regions with high image homogeneity, and aid in mitigation of SAR deposition (Padormo et al., 2015). Currently, only one pTx head coil with 8-transmit elements and 32-receive channels is approved for clinical 7T imaging (Nova Medical Inc., USA), although many bespoke research coils have been designed (Williams et al., 2023).

In theory, 7T MRI is suited for highly accelerated parallel imaging because the inherent higher SNR can tolerate the SNR penalties associated with large acceleration factors (Wiesinger et al., 2004, 2006; Ugurbil et al., 2019). In addition, the greater spatial variation in the receive sensitivity profiles at 7T can be exploited to improve parallel imaging. High acceleration is functionally critical at 7T. Without it, the scan times required to fully realize sub-millimeter resolution become clinically impractical. Moreover, long readout echo trains in the absence of acceleration exacerbate ${\text{T}}_{2}^{*}$-induced blurring, which is more severe at 7T due to shorter intrinsic ${\text{T}}_{2}^{*}$ values. Lastly, parallel imaging also reduces echo spacing in EPI sequences, helping to mitigate geometric distortion and signal pile-up caused by increased B_0_ inhomogeneity at high field. As such, high parallel imaging performance is a key enabling factor for translating the theoretical resolution and contrast benefits of UHF imaging into clinically usable protocols.

However, these advantages cannot be fully exploited because although 64-channel receive coils are commercially available and routinely used at 3T, equivalent high-channel-count systems for 7T remain limited in availability (Ugurbil et al., 2019; Mareyam et al., 2020; May et al., 2022; Gruber et al., 2023; Feinberg et al., 2023). This hardware gap restricts the maximum achievable acceleration, meaning that many of the theoretical gains of 7T imaging are difficult to realize consistently in clinical settings and it represents a missed opportunity at this stage of UHF adoption.

At the University of Glasgow, we developed an eight-channel transmit 64-channel receive (8Tx64Rx) coil in collaboration with NHS GGC and MR CoilTech Limited. An *in vivo* study was conducted in a clinical setting with healthy volunteers to evaluate and assess its diagnostic quality with respect to the commercial single-channel transmit 32-channel receive (1Tx32Rx) regulatory approved 7T head coil. This paper reports the findings of this study. Two prototypes of the coil were designed and constructed during the project. Both prototypes were electrically equivalent, however, the second prototype incorporated refinements in the coil housing design to improve its robustness.

## 2 Materials and methods

### 2.1 Coil design and construction

To address the constraints of clinical imaging at 7T, a head coil consisting of 8Tx channels in combination with 64Rx channels was designed. The transmit array consist of a novel, nested arrangement of conventional loop transmit elements to minimize mutual inductive coupling between the adjacent and second neighboring elements (Williams et al., 2021). A local RF shield was placed around the transmit array to minimize radiation loss due to wave propagation in the scanner bore at 300 MHz (Brunner et al., 2009). Patient comfort was enhanced by an open-face design approach enabled by two large cut-outs in the RF shield. This allows a look-out mirror to be fitted outside the transmit coil for the subjects to see the operator during the scan, similar to conventional clinical 1.5T/3T head coils.

To improve SNR and accelerated imaging performance, the receive array was equipped with 64-receive channels while the current industry standard 7T head coil consists of a 32-channel receive array. The 64Rx elements were arranged in a split-top housing consisting of an anterior half with 24-channels and a posterior half with 40-channels. The receive helmet was designed based on an anthropometric study to enable the best fit for different head, and neck shapes. To improve patient comfort and operator handling, a sliding mechanism was implemented, which allowed the transmit array and the anterior half of the receive array to be moved back and forth and locked in place. Coil housing design focused on patient and operator comfort, reducing feelings of claustrophobia and minimizing coil weight for an efficient workflow.

**Figure 1:**
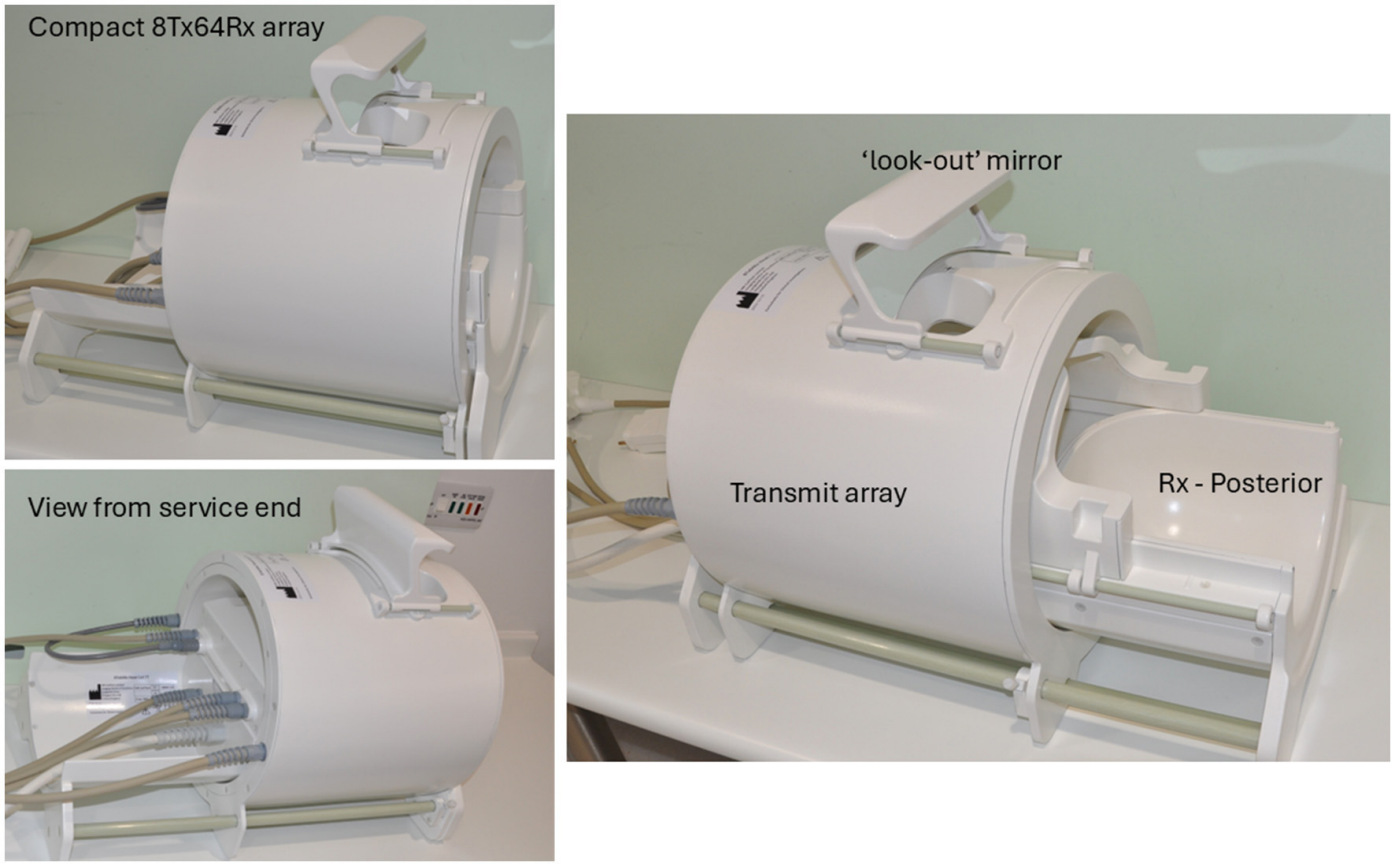
Photograph of the completed 8Tx64Rx 7T head coil as viewed from the front and service end. The transmit array and anterior half of the receive array slides back and forth on the sliding rods. A detachable “look-out” mirror is mounted on the transmit array.

The electrical design was based on our previously described open-face 8-channel transmit array design, which was initially combined with a 32Rx receive array (Williams et al., 2021). To drive the transmit array in circularly polarized (CP) mode, a phase increment of 45° was applied to the adjacent channels. A photograph of the completed coil is shown in Figure 1. RF coil layout and internal photographs can be found in (Williams et al. 2021), (Gunamony and Feinberg 2022), and (Feinberg et al. 2023).

### 2.2 Coil performance testing and safety validation

#### 2.2.1 Benchtop evaluation of coil

To characterize the coil in the lab, test rigs were custom-built to mimic the scanner control signals for switching and biasing of the PIN bias lines and biasing the preamplifiers. Bench measurements were performed using a two-port ZND series vector network analyser (Rohde and Schwarz, Germany). The S-parameters of the transmit and receive arrays were measured by loading the coil with a head-and-shoulder phantom filled with tissue equivalent solution (ε_*r*_ = 52.1, σ = 0.41 S/m). Final tuning of the transmit array was performed in the presence of the actively detuned receive array.

#### 2.2.2 Electromagnetic field simulation and SAR management

The 3D electromagnetic (EM) and circuit co-simulation was carried out using the time domain solver CST Studio suite 2021 (Dassault Systems, Paris, France), which uses the finite-integration technique. The simulation included coil conductors (copper wire, conductivity = 5.8 × 10^7^ S/m) as well as capacitors with series resistance from the component datasheet, which were modeled as lumped elements in the 3D simulation. The local RF shield, coil housing and the scanner bore were also included in the model. The fiberglass was standard FR-4 from the CST database: permittivity, ε = 4.3 F/m; relative permeability, μ = 1; electrical loss tangent, tan(δ) = 0.025. The coaxial cables, cable traps and wiring were not considered in the simulation. The variable capacitors, matching circuit and decoupling inductors were represented as ports resulting in a total of 34 ports in the 3D EM simulation. There were eight excitation ports in the circuit co-simulation, and each one was connected to the matching network via an attenuator to represent the cable loss from the coil feed-point to the coil plug. The RF shield included the open-face cut-out in the shape of goggles as implemented in the mechanical CAD model.

**Figure 2:**
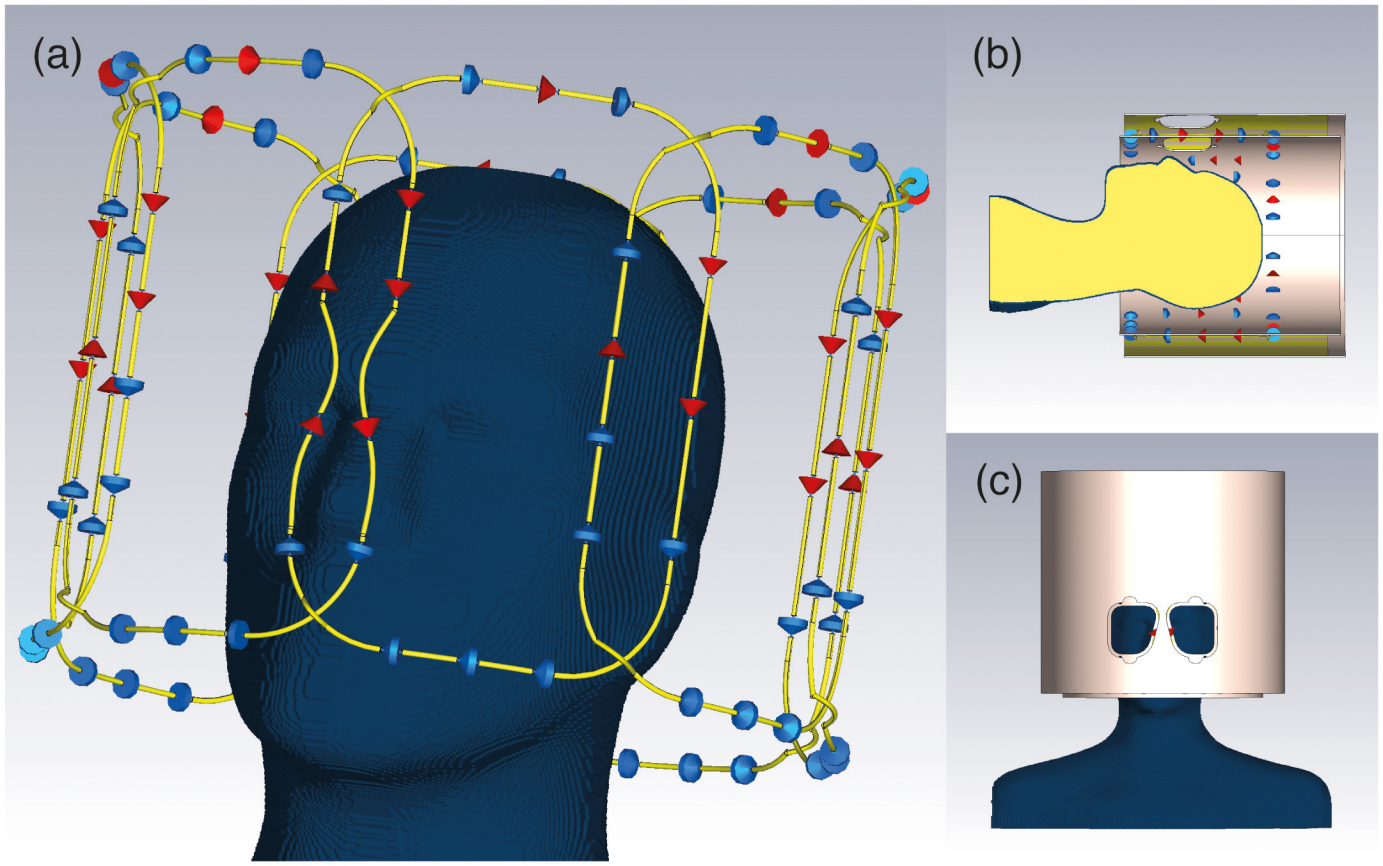
Simulation model **(a)** including copper wires (yellow), ports (red cones, including variable components and decoupling inductances) and fixed capacitors (blue cones). The RF shield modeled with eye cut-outs is shown on the right top **(b)** and including the coil housing right bottom **(c)**. The gradient shield is also included in the model, but is not shown in this figure.

A voxel model of the head-and-shoulder (HS) phantom with 1 mm isotropic resolution was imported from a CT scan. The electrical properties of the phantom solution were measured and applied to the simulation. The coil was then tuned and matched to this HS phantom at 297.2 MHz with 50 Ω in the circuit co-simulation. The positioning of the HS phantom with the EM simulation of the transmit array and shield is shown in Figure 2.

**Figure 3:**
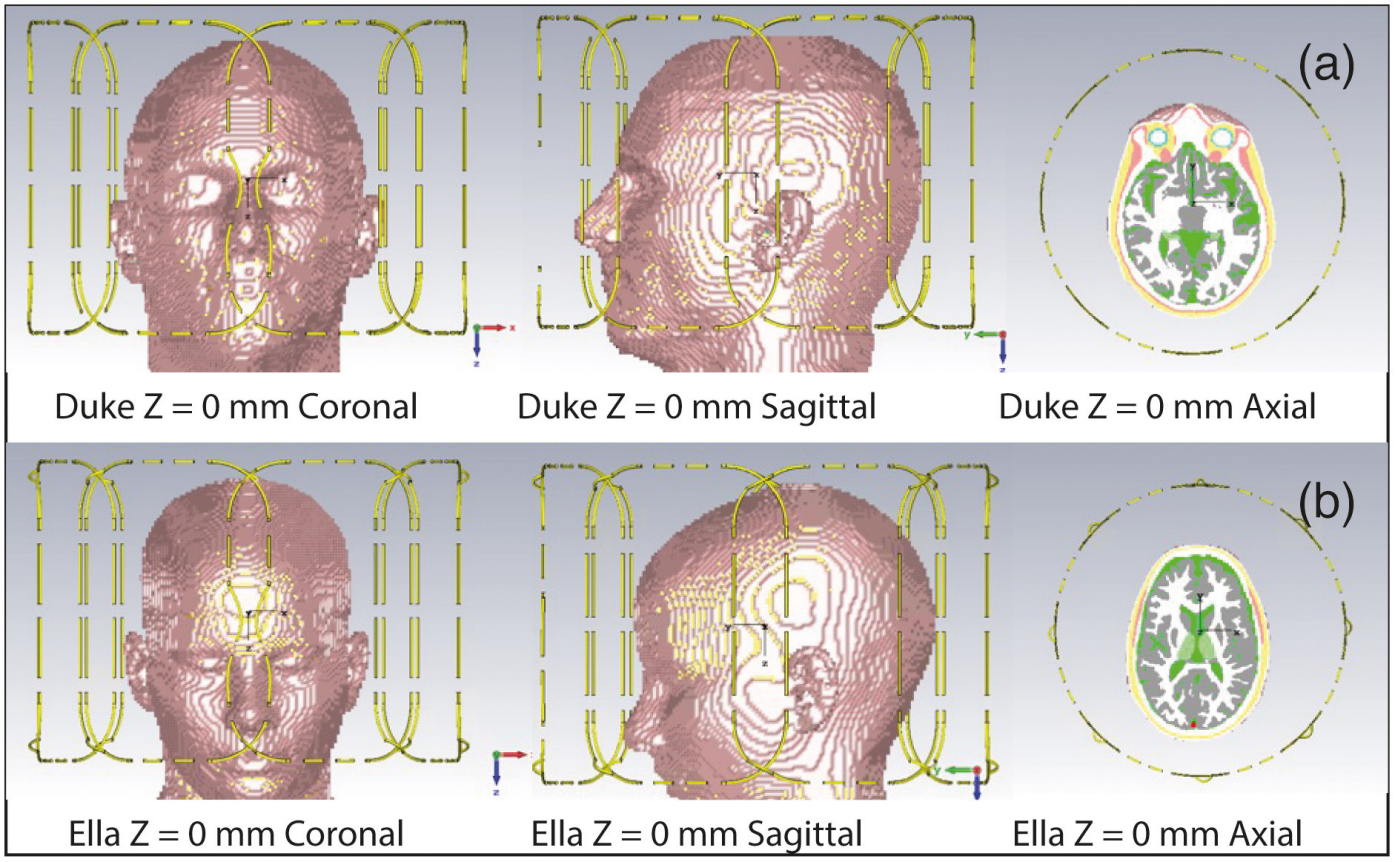
Position of human body models relative to the transmit coil at the reference position. Duke is shown in **(a)** and Ella in **(b)**.

The coil was then loaded with the Duke and Ella models from the Virtual Family cohort (Gosselin et al., 2014), simulated at a total of 14 positions with the coil and truncated at the level of the chest. The EM field and SAR were simulated for both models at different positions (seven-each) to represent the different possible subject positions during scans *in vivo*. The isocenter positioning of both body models in the transmit array simulation is shown in Figure 3. A typical mesh consisted of about 40 million cells. The frequency sweep was set from 280 to 320 MHz, and a convergence criterion of −40 dB was set to obtain the RF field distribution for each discrete port. On a dual Intel Xenon workstation with 256 GB RAM and GPU acceleration using Nvidia Tesla K80, simulation time for each body model was just over a day.

Q-matrices (Sbrizzi et al., 2012; Graesslin et al., 2012) were exported from CST, concatenated together, and compressed into a final set of virtual observation points (VOPs) for local SAR management on the scanner using the compression method of Eichfelder and Gebhardt, 2011. Using a 25% overestimation of the worst case vector SAR, 29 VOP matrices were produced in compression. These final VOPs was scaled to account for intersubject variability, difference between simulation and measurement and the scanner manufacturer recommended measurement system error. SAR monitoring during non-pTx or CP operation is managed with a “*k*”-factor set in the coil file.

#### 2.2.3 Thermometry for electromagnetic simulation validation

Measurements of RF heating were performed with a 3.5 liter lightbulb-shaped phantom filled with a PVP, NaCl, Agar solution (σ = 0.495 S/m, ε_*r*_ = 66; Ianniello et al., 2018; Brink et al., 2018). The proton resonance frequency shift method (PRFS) was used to calculate maps of the temperature change based on the phase difference in 3D GRE sequence (acquired coronally, 3.5 mm isotropic resolution, TE 8 ms, TR 24 ms, Bandwidth per pixel = 555 Hz, TA 1 min) before and after RF heating of the phantom (De Poorter et al., 1995; Oh et al., 2013). A PRF change coefficient of α = −0.01 ppm/°C was used.

The measurement of RF heating was complicated by an additional temperature increase caused by the electronics of the receive coil. To correct this, prior to the thermometry measurement, two 3D GRE sequences were acquired 20 min apart, and a map of the temperature increase was measured using the PRFS method (Boulant et al., 2015). This was subtracted from the measurement with RF heating.

The RF heating experiments were performed in CP mode with a power of 14.3 W per channel at the coil plug over 20 min. A fiber optic temperature probe (Osensa Innovations, Canada) was used to measure the temperature at a point in the phantom for calibration.

For simulated temperature maps, the EM simulations were again performed in CST software and exported on a 5 mm isotropic and cartesian grid. Post-processing was performed in MATLAB by calculating SAR, followed by integration of the heat diffusion equation with the above parameters of the phantom (Hoffmann et al., 2015). This model assumes the energy is deposited instantaneously. Heat dissipation over time was not modeled in the simulation.

### 2.3 Coil validation and neuroimaging study

#### 2.3.1 Study design

Project-specific Research Ethic Committee approval was obtained for the study (MVLS College Ethics Committee, University of Glasgow, UK; REC Reference Number 200210019). The study was conducted jointly between the University of Glasgow and NHS Greater Glasgow and Clyde (NHS GGC) under the supervision of the NHS GGC Research and Development Clinical Research Facility according to established Standard Operating Procedures.

Healthy volunteers (HVs) with no contraindication to MRI and no known brain pathology were recruited from an NHS GGC healthy volunteer bank. All study procedures occurred after the participants provided informed consent, according to Good Clinical Practice (GCP) research standards.

Fifteen healthy volunteers were initially recruited, of which 13 of them (eight females, five males; age range 18–75 years with an average age of 42.5 years) completed the study. One volunteer dropped out after one session due to scheduling issues while the other volunteer was severely claustrophobic and the scan was aborted after an initial localizer.

All volunteers underwent three scanning sessions on separate days on a MAGNETOM Terra 7T MRI system (Siemens Healthineers, Erlangen, Germany). One session was with a widely used 1Tx32Rx head coil (Nova Medical, USA), and the other two sessions were with our home-built 8Tx64Rx coil, once in CP mode and once using pTx to distinguish the contributions from increased receive channel count and pTx. All sessions were without the use of dielectric pads. The order of scans was randomized by a statistician prior to starting recruitment, and the HVs were not informed of the specific coil type, coil design, or scanning mode used in each session.

The following parameters were analyzed for performance assessment and validation of the coil as a potential clinical tool: participant and operator questionnaires were used to assess comfort and usability, radiology scoring by experienced radiologists on image quality, artifacts, homogeneity in various brain regions, and quantitative image homogeneity assessment. The endpoint was to demonstrate potential clinical usability and benefits of our home-built 8Tx64Rx coil; and to evaluate if it has equivalent or improved performance compared to the standard 1Tx32Rx head coil.

#### 2.3.2 Neuroimaging sequences

For each session, after the initial localiser, a combined transmit ${\text{B}}_{1}^{+}$ map was acquired using a saturation-prepared ultrafast spoiled gradient echo technique (Siemens' “tfl_rfmap”). This was followed by structural imaging, including 3D T_1_-weighted (T_1_w), 2D T_2_-weighted (T_2_w) Turbo-Spin Echo (TSE,) 2D T_2_ Fluid-Attenuated Inversion Recovery (FLAIR), and 2D Proton Density (PD)-weighted TSE. MR angiography of the CoW was performed using a TOF-MRA sequence, and SWI was also performed. Lastly, diffusion weighted imaging (DWI) was performed using a multishot readout segmented spin-echo EPI sequence (“RESOLVE”). Detailed sequence parameters are given in Table 1.

**Table 1:** Imaging parameters used across all scans.

MR Sequence	T_1_w FLASH	T_2_w TSE	T_2_ FLAIR	PDw TSE	SWI	TOF MRA	DWI RESOLVE
TA (min)	9:17	3:18	4:03 (x2)	3:55	7:27	3:52 (x2)	3:43
TR (ms)	35	9,000	12,820	5,000	21	13	4,930
TE (ms)	2	58	83	9.7	14	4.58	58/86
TI (ms)	–	–	2,600	–	–	–	–
FA (°)	50	135	135	135	10	20	180
2D/3D	3D	2D	2D	2D	3D	3D	2D
FOV (mm^3^)	225 × 240 × 225	172 × 230 × 148.2	172 × 230 × 148.2	172 × 230 × 148.2	178 × 220 × 127.8	150 × 210 × 56.26	229 × 229 × 151.2
Matrix size	300 × 320 × 299	768 × 1,024 × 39	672 × 896 × 39	768 × 1,024 × 39	1,456 × 1,792 × 72	506 × 704 × 158	224 × 224 × 39
Resolution (mm^3^)	0.75 iso	0.22 × 0.22 × 3	0.26 × 0.26 × 3	0.22 × 0.22 × 3	0.12 × 0.12 × 1.5	0.3 × 0.3 × 0.3	1 × 1 × 3
Slice/slab distance factor (%)	–	30	30	30	20	−19.44	30
Parallel imaging (GRAPPA factor)		2	2	2	3	3	3
Partial Fourier		No	No	No	No	7/8	No
No. averages	1	1	1	1	1	1	1
Turbo factor	–	9	9	3	–	–	(7 RO segments)
Bandwidth (Hz/Px)	475	285	285	285	205	205	700
Grad./RF		Fast/low SAR	Fast/low SAR	Fast/low SAR	Normal/normal	Fast/spoil	Fast/normal
${\text{B}}_{1}^{-}$ receive profile correction	B_1_ filter	B_1_ filter	B_1_ filter	B_1_ filter	–	B_1_ filter	–
pTx method	Volume ${\text{B}}_{1}^{+}$ shim	DiSCoVER dynamic shim	None	DiSCoVER dynamic shim	None	None	Slice-specific ${\text{B}}_{1}^{+}$ shimming

In addition to the imaging sequences, several additional calibration measurement scans were performed at the start of each subject scan: advanced static field B_0_ shimming (Aghaeifar et al., 2020) (using a works-in-progress package #1441, Siemens Healthcare Ltd., Camberley, UK), and relative, single-channel transmit field ${\text{B}}_{1}^{+}$ mapping using the vendor-provided Presaturated TurboFLASH sequence (Chung et al., 2010). In total, these initial calibration scans accounted for less than 5 additional minutes of scan time. These calibration measurements were performed once at the start of the examination and reused across all subsequent pTx sequences, such that no additional per-sequence calibration scans were required.

For each imaging sequence, the pTx approach was selected based on the technical considerations of each sequence type. For the T_1_-weighted 3D FLASH sequence, a static volumetric ${\text{B}}_{1}^{+}$ shim was applied using the scanner's in-house optimization routine. For the T_2_- and PD-weighted TSE scans, the dynamic volumetric ${\text{B}}_{1}^{+}$ shimming method DiSCoVER (Malik et al., 2014; Tomi-Tricot et al., 2021) was used with the manufacturer works-in-progress #1443B (Siemens Healthcare Ltd, Camberley, UK). For the readout-segmented diffusion imaging, an in-house developed slice-by-slice shimming routine was used (Ding et al., 2024). The remaining three contrast types did not offer a pTx variant at the time of the study due to sequence local SAR restrictions (in the case of T_2_ FLAIR and TOF MRA) and/or lack of available programmable sequence for modification (TOF MRA and SWI). The computational overhead associated with pTx optimization varied by sequence type but was generally low: volumetric shimming was performed automatically on the scanner with negligible delay, DiSCoVER required on the order of tens of seconds, and slice-by-slice shimming was performed offline during acquisition of other sequences and, therefore, did not increase overall scan time. While these additional steps introduce some workflow complexity, the overall time overhead was modest.

#### 2.3.3 Evaluation of operator experience

Ease of handling and use of the 8Tx64Rx coil was assessed in both CP and pTx, modes, and compared to the standard 1Tx32Rx head coil. An in-house designed questionnaire was used (Supplementary Figure S1) and scores were captured on a five-point Likert ordinal scale. Questions scored included: friendliness of the user interface; handling and moving of the coil; positioning of the coil in the scanner; positioning of the person to be scanned in the field of view; protocol handling during scanning; ease of communication with the person being scanned; overheating of parts leading to termination of scan; overall ease of scan. Each of the parameters were scored on a scale from 1 to 5, with “1” being “very unhappy” and “5” being “highly satisfied.” This was scored by eight different operators (radiographers and physicists, some with limited prior experience scanning with the 8Tx64Rx coil and using pTx techniques) reflecting their overall assessment and experience during the scanning of the 13 HVs.

#### 2.3.4 Evaluation of participant comfort and experience

HVs were given a locally designed questionnaire (Supplementary Figure S2) to score and capture their experience, comfort and acceptability of the scan, using a five-point Likert ordinal scale. Questions asked included: How comfortable was the scan? How comfortable was the head coil? Did you feel claustrophobic? Did the coil make contact with the scanning team easy? If you were a patient, would you be happy to come for this type of examination? Further, ranking of the overall comfort of the scanning session, “Please rank the scanning session on overall comfort of the scan,” was scored using a 10-point Likert ordinal scale.

#### 2.3.5 Evaluation of neuroimaging sequences

All images obtained were scored to assess overall image quality, and diagnostic quality, with the scoring radiologist blind to the type of coil and the imaging mode the images were obtained with. Further, for scoring purposes, the brain was “segmented” into three areas (1. superior frontal/parietal/occipital lobes; 2. temporal lobes; 3. posterior fossa), and scored independently for each sequence (3D T_1_w, T_2_w, T_2_ FLAIR, PD, DWI, SWI, and TOF). The sequences were scored based on their image quality, degree of artifact, signal homogeneity, contrast and overall diagnostic value. An ordinal Likert scale was used, with scores ranging from “1,” “poor” to “5,” “excellent.” The “Radiology Questionnaire” used is presented in Supplementary materials as “Supplementary Figure S3.” Scans were assessed as to the presence of any significant motion artifact.

All scans were scored three times by different, independent experienced neuroradiologists or radiologists with neuroradiology subspecialty interest. The lead neuroradiologist and study principal investigator (PI) scored all scans; further four radiologists scored half of the scans each. The radiologists involved either had 7T expertise and prior study experience, including scoring of scans, or were familiar with 7T images. All images were blinded before scoring.

#### 2.3.6 Quantitative analysis of image homogeneity

Quantitative evaluation of image homogeneity was carried out using the DWI b = 1000 s/mm^2^ maps and T_2_ FLAIR images in view of their dependence on B_1_ effects at UHF, in addition to T_2_w TSE images. The images were first blinded, before 10 circular ROIs were placed in the left and right cerebellum, as well as in the left and right temporal, frontal, occipital and parietal lobes. The mean signal was recorded for each ROI.

#### 2.3.7 Statistical analysis

All scores were unblinded prior to statistical analysis, which was performed using Python (v3.13). For each coil configuration, individual ratings were first averaged across raters (HVs, operators, or radiologists). Scores were then averaged across subjects and/or regions of interest for each assessed parameter, where applicable, and statistical testing was performed on these aggregated mean scores.

A Kruskal–Wallis test was used to determine any significant differences between coils in data derived from the patient questionnaire, operator questionnaire, and radiology scoring. These measures were based on Likert-scale ratings and therefore treated as ordinal data, for which non-parametric testing is appropriate, particularly given the observed skew toward higher scores. Where the overall test was statistically significant, *post-hoc* pairwise comparisons were performed using Dunn's test with Holm correction for multiple comparisons.

For quantitative analysis of image homogeneity, the coefficient of variation across all 10 ROIs (standard deviation of the mean signal across ROIs divided by the average mean signal) was calculated for each subject and compared across the three coils. Pairwise Student's *t*-tests were performed.

Across all data, no anomalous data was excluded and statistical significance was defined as *p* < 0.05.

### 2.4 Evaluation of SNR and g-factor performance

An additional study was conducted to compare the SNR and parallel imaging performance of the coil against the reference coil. Three healthy volunteers were scanned on the same 7T MRI scanner with the commercial 1Tx32Rx coil and with the custom-built 8Tx64Rx coil. Scanning took place over two sessions on separate days, and the order of scanning was randomized.

For each volunteer, combined ${\text{B}}_{1}^{+}$ maps were acquired axially using actual flip angle imaging (Yarnykh, 2007) with the following parameters: TR = 10/100 ms, TE = 2.04 ms, flip angle = 60°, resolution = 4 mm^3^ isotropic, FOV = 240 × 240 mm^2^ with 72 slices, GRAPPA acceleration factor = 3 (32 reference lines).

SNR maps were acquired using a non-accelerated 2D GRE sequence with the following parameters: TR = 8,000 ms, TE = 3.82 ms, flip angle = 40°, resolution = 4 × 4 × 3.2 (slice) mm^3^ with 25% slice gap, FOV = 240 × 240 mm^2^ with 72 slices, imaging bandwidth = 990 Hz/Px. A noise scan was subsequently acquired using matching parameters, except the TR was changed to 500 ms, and the transmit voltage was set to 0 V.

Structural imaging was performed sagittally using a T_1_-weighted MP2RAGE sequence at 0.75 mm^3^ isotropic resolution [TR = 5,000 ms, TE = 1.92 ms, TI = 700/2,700 ms, flip angles = 4/5°, GRAPPA acceleration factor = 5 (32 reference lines)].

SNR and g-factor maps were reconstructed offline in MATLAB (R2023b, Mathworks, USA) using raw k-space data following the methodology of (Lakhani et al. 2022); (Kellman and McVeigh 2005); (Kellman 2007). First, the noise covariance was estimated from the noise-only data and scaled by the noise bandwidth. Afterwards, the phased array combined image reconstruction was performed using the root-sum-of-squares equation from Roemer et al., 1990 to obtain the SNR-scaled images. The g-factor maps for eight different SENSE acceleration factors were computed using the approach of Pruessmann et al., 1999. The SNR and g-factors maps were registered to MNI152 standard space via the base GRE and the high-resolution T_1_-weighted MP2RAGE structural image images using FSL (FMRIB Software Library) registration tool, FLIRT.

Two regions of interest, “periphery” and “central,” were defined for performance comparison. All statistical testing was done with paired Student's *t*-tests without excluding anomalies.

## 3 Results

### 3.1 Benchtop evaluation of coil

**Figure 4:**
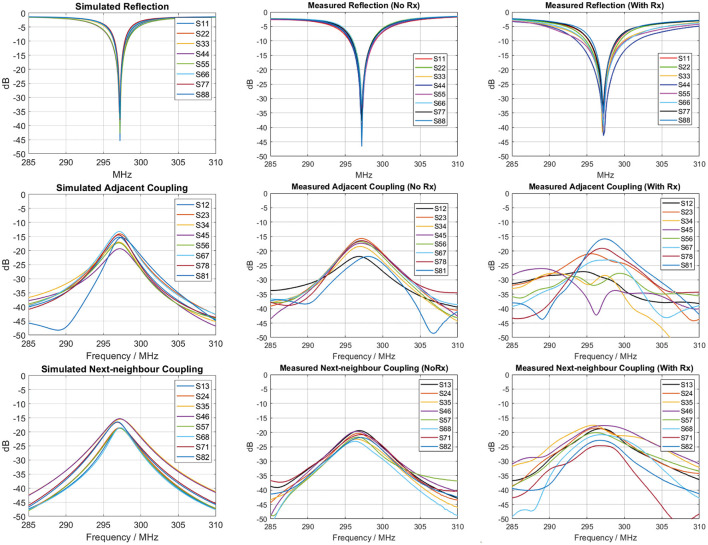
Comparison of S-parameters between simulation (left column) and measurements (middle column: without Rx; right column: with Rx). **(Top)** reflection for each channel; **(Middle)** coupling to adjacent element; **(Bottom)** coupling to next neighboring element. The coil is loaded with the head and shoulder phantom both simulation and measurement.

The simulated and measured S-parameter plots of the tune and match, adjacent element, and second-neighboring element coupling of the constructed transmit array with and without the receive array are shown in Figure 4. Note that the simulated S-parameters are shown without the receive array because the numerical model does not include the receive array.

### 3.2 Safety validation

#### 3.2.1 Simulated and measured ${\text{B}}_{1}^{+}$ map

**Figure 5:**
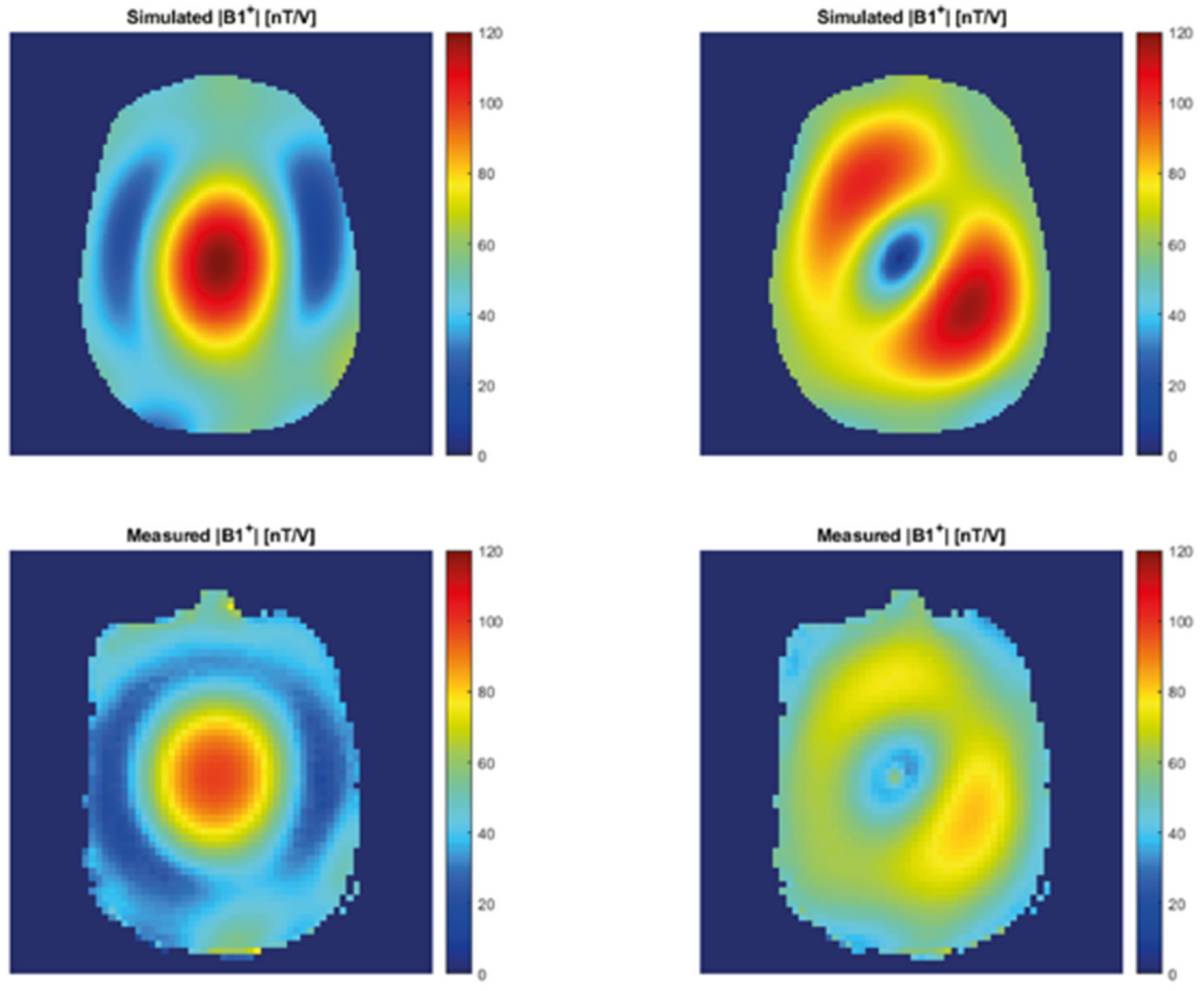
CP **(left)** and CP2+ **(right)** ${\text{B}}_{1}^{+}$ field maps of the central axial slice of the transmit array. Upper row shows the simulated maps and lower row shows the measured ${\text{B}}_{1}^{+}$ maps. The losses are referenced to the coil plug.

The simulated and measured ${\text{B}}_{1}^{+}$ maps in the HS phantom are shown in Figure 5. In CP mode, the peak |${\text{B}}_{1}^{+}$| in the center of the phantom was 120 nT/V in simulation and 98.1 nT/V in experiment. The average ${\text{B}}_{1}^{+}$ in the central axial slice after filtering out the low flip angles was 81.9 nT/V and 71.1 nT/V in simulation and measurement, respectively. The cable losses are calibrated, and these values are referenced to the coil plug. In CP^2+^ mode, the peak |${\text{B}}_{1}^{+}$| was 116.7 nT/V in simulation and 82 nT/V in experiment. The differences between simulation and experiment can be attributed to the attenuation of the transmit field due to the shielding effect of the 64-channel receive electronics. In addition, modeling inaccuracies include variable capacitors defined as ideal components due to non-availability of data and resistive losses from solder joints. Previously, we reported a model of a 64Rx array inside an 8-channel transmit array showing a 10% attenuation of the ${\text{B}}_{1}^{+}$ (McElhinney and Gunamony, 2022).

#### 3.2.2 Simulated and measured temperature maps

**Figure 6:**
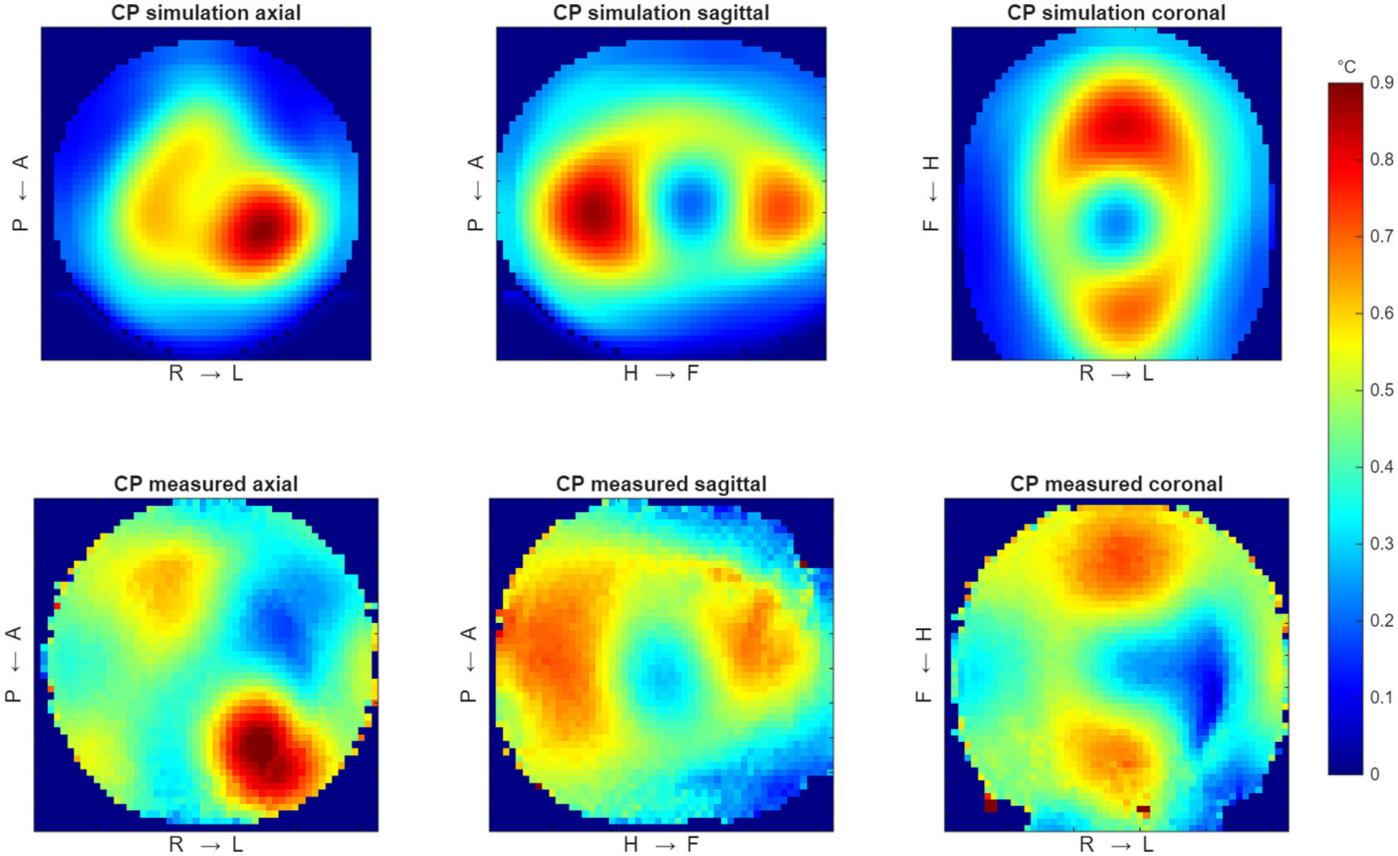
Comparison of simulated **(top)** and measured **(bottom)** temperature maps in CP mode.

The simulated temperature rise and experimental temperature rise measurements in a phantom using the PRFS method are shown in Figure 6 for CP mode. The measurement and simulations show reasonable agreement despite minor discrepancies attributed to modeling limitations and duration of the experiment.

#### 3.2.3 Receive coil heating

During scanning, heating of the RF receive coil housing was observed due to the dense electronics and preamplifiers of the 64Rx channels. A coil housing surface temperature rise of 5°C after 20 min of scanning at 100% SAR was measured with an external temperature camera. This heating of the receive coil housing also caused complications in the temperature mapping validation of RF heating, because the temperature rise due to the receive coil is greater than the RF-induced temperature rise. The effect of the receive heating can be seen in Supplementary Figure S4.

### 3.3 Coil validation and neuroimaging study

#### 3.3.1 Operator experience

**Figure 7:**
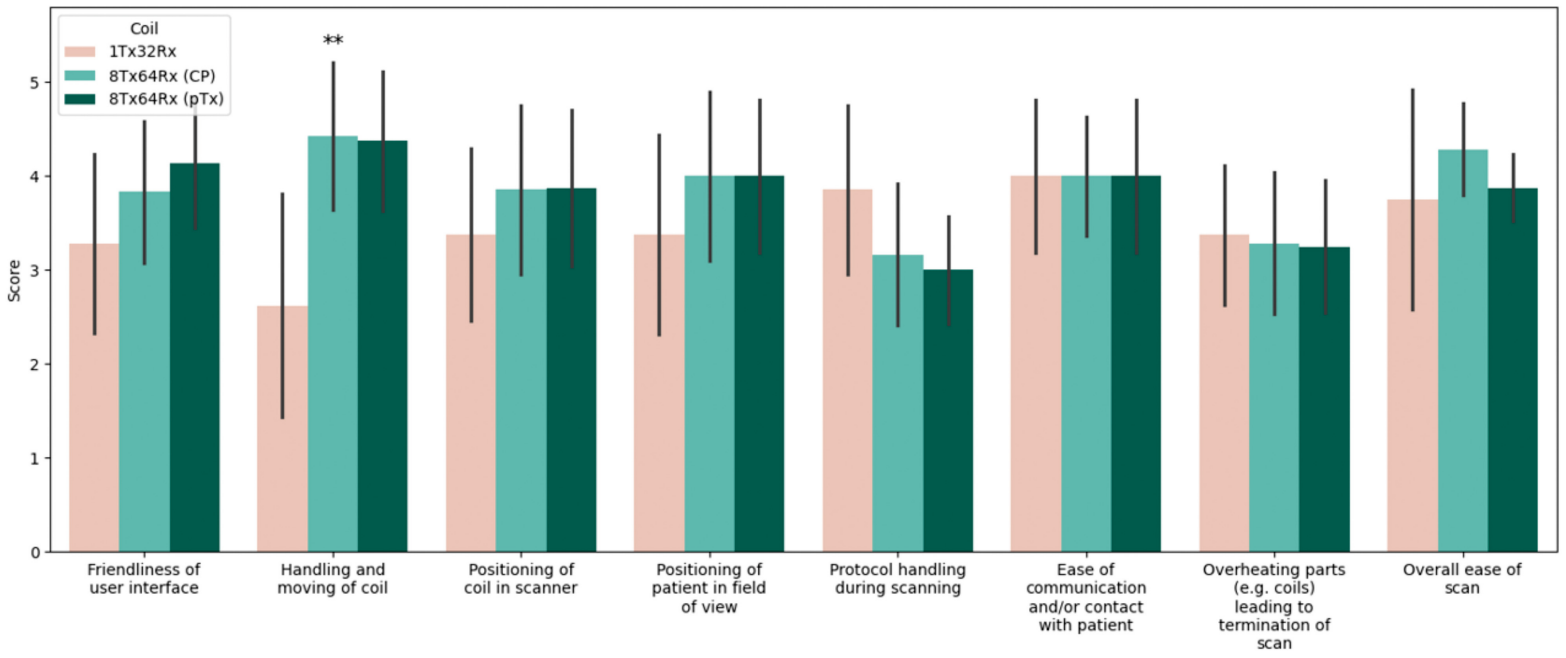
Bar graphs comparing various parameters relating to operator handling and assessment of the 1Tx32Rx coil, and the 8Tx64Rx coil used in CP and pTx mode. Data are presented as mean ± standard deviation. Statistical significance between coil configurations is indicated by asterisks (**P < 0.01).

Figure 7 shows a bar graph of the operator assessment. Across the eight parameters assessed, only one parameter, handling and moving of the coil, showed significant differences across the three sessions. The mean scores for handling and moving of the coil were 2.63 ± 1.19 for 1Tx32Rx, 4.38 ± 0.74 for 8Tx64Rx (CP), and 4.43 ± 0.79 for 8Tx64Rx (pTx). *Post-hoc* Dunn tests showed significant differences between 1Tx32Rx and 8Tx64Rx (CP), and between 1Tx32Rx and 8Tx64Rx (pTx) coils (*p* = 0.019 for both).

All other assessed parameters showed no statistically significant differences between coils. There was also no statistically significant difference in operator scores between CP and pTx modes for the 8Tx64Rx coil.

#### 3.3.2 Participant experience and comfort

**Figure 8:**
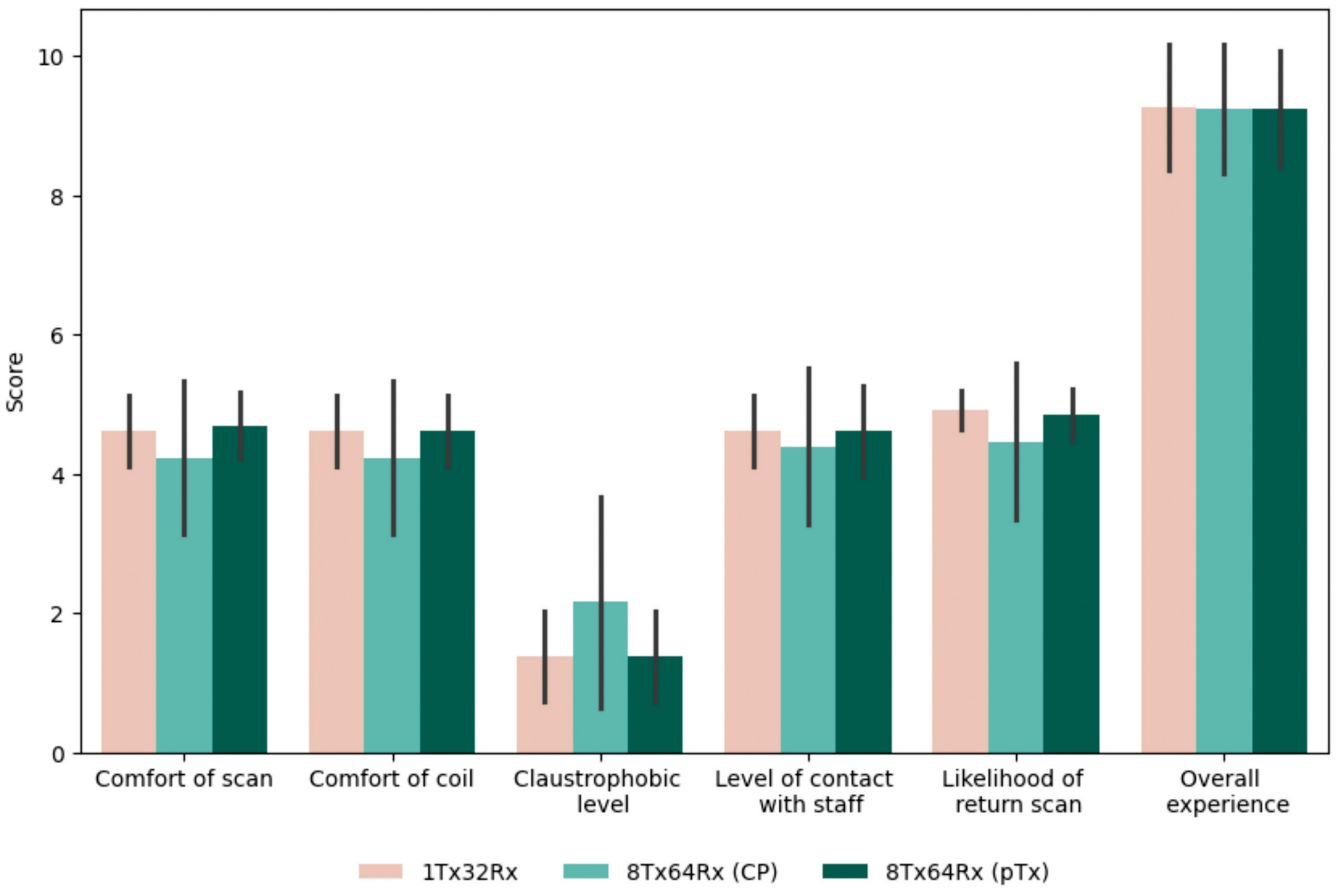
Mean patient experience questionnaire scores across all assessed parameters for three coil configurations [1Tx32Rx, 8Tx64Rx (CP), and 8Tx64Rx (pTx)]. Data are presented as mean ± standard deviation. No statistically significant differences were found between the three scans. Note that the first five assessed parameters were scored out of five, while the last parameter, overall experience, was scored out of 10.

Figure 8 shows bar graphs capturing patient experience, and Table 2 summarizes mean values and standard deviations. There were no statistically significant differences between the coils, with overall positive scores for all coils and scanning modalities.

**Table 2:** This shows mean values and standard deviations for the various parameters assessed for participant comfort.

Parameter assessed	1Tx32Rx	8Tx64Rx (CP)	8Tx64Rx (pTx)	*P*-value
Comfort of scan	4.62 ± 0.51	4.23 ± 1.10	4.62 ± 0.51	0.41
Comfort of coil	4.62 ± 0.51	4.23 ± 1.10	4.62 ± 0.51	0.57
Claustrophobia	1.38 ± 0.65	2.15 ± 1.52	1.38 ± 0.65	0.27
Level of contact with staff	4.62 ± 0.51	4.38 ± 1.12	4.62 ± 0.65	0.92
Likelihood of return	4.92 ± 0.28	4.46 ± 1.13	4.85 ± 0.38	0.28

#### 3.3.3 Evaluation of Neuroimaging Sequences

No scans had to be excluded from analysis due to significant motion artifact. Across all sequences, the overall image quality averaged 3.43 ± 0.64 for 1Tx32Rx, 3.59 ± 0.60 for 8Tx64Rx (CP), and 3.82 ± 0.25 for 8Tx64Rx (pTx); while the overall diagnostic quality was 3.35 ± 0.70 for 1Tx32Rx, 3.59 ± 0.55 for 8Tx64Rx (CP), and 3.83 ± 0.35 for 8Tx64Rx (pTx). While mean ratings for both overall image quality and overall diagnostic quality were numerically highest for the 8Tx64Rx (pTx), followed by the 8Tx64Rx (CP), and lowest for the 1Tx32Rx coil, there were no statistically significant differences in either parameter across the three coils (with *p* = 0.13 for diagnostic quality, and *p* = 0.26 for image quality).

**Figure 9:**
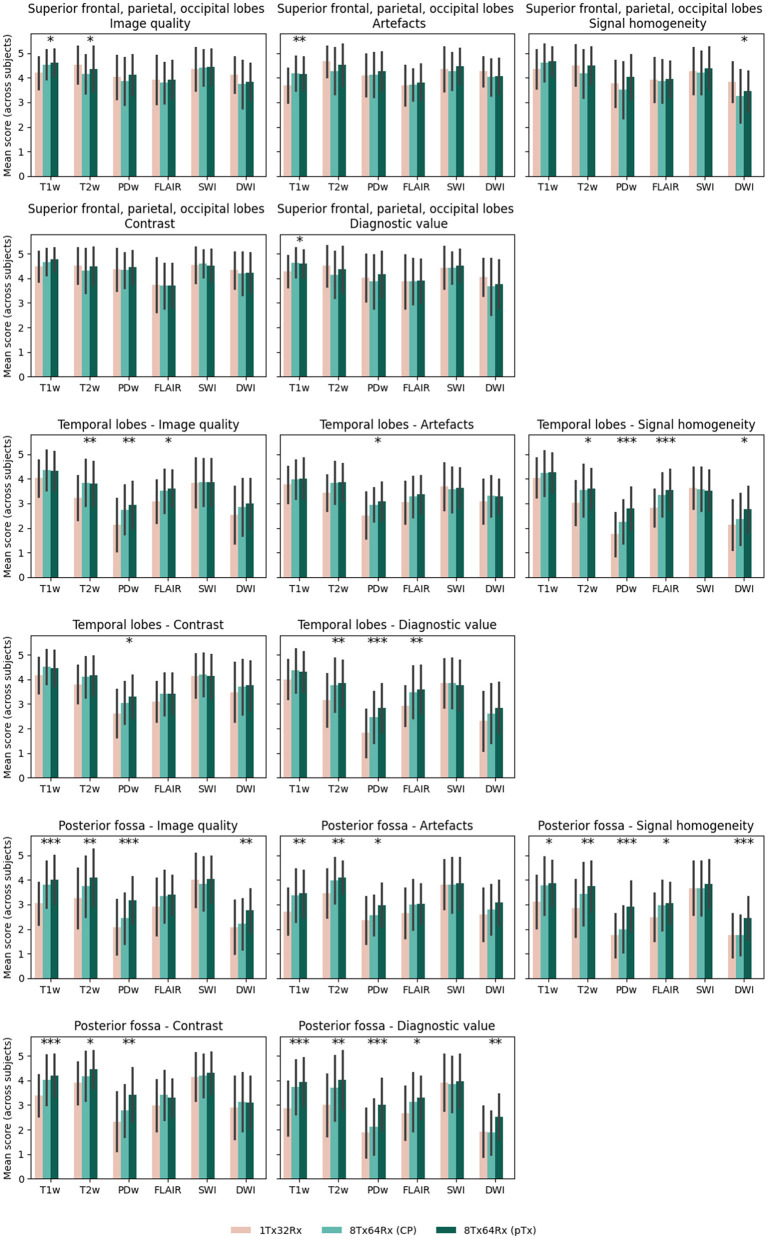
Mean radiological assessment scores for image quality, artifacts, signal homogeneity, contrast, and diagnostic value across brain regions and sequences for three coil configurations [1Tx32Rx, 8Tx64Rx (CP), and 8Tx64Rx (pTx)]. Scores represent the mean rating averaged across three radiologists. Error bars indicate standard deviation across subjects. Statistical significance between coil configurations is indicated by asterisks (*P < 0.05, **P < 0.01, and ***P < 0.001).

Figure 9 compares the image quality, artifacts, signal homogeneity, contrast and diagnostic value in the superior frontal, parietal, occipital lobes, the temporal lobes, and the posterior fossa across T_1_w, T_2_w, PDw, T_2_ FLAIR, SWI, and DWI. The ToF MRA has been performed with a slab encompassing the CoW, with hence other brain areas not assessed. The complete table of results is given in Supplementary Table S1 in the supporting information.

Across all assessed parameters and anatomical regions, both 8Tx64Rx configurations (CP and pTx) demonstrated equivalent or superior performance compared with the 1Tx32Rx coil, with the exception of signal homogeneity in DWI within the superior frontal, parietal, and occipital regions. Regional comparison showed the greatest improvement within the posterior fossa, followed by the temporal lobes, whereas performance differences between coils were less pronounced within the superior frontal, parietal, and occipital regions.

The most significant improvements are seen scanning with the 8Tx64Px coil in either mode compared to the 1Tx32Rx coil at the level of the posterior fossa; with significant difference also between scanning using the 8Tx64Px coil in pTx compared to CP mode. This affects T_1_-weighted imaging, T_2_ TSE, PD, T_2_ FLAIR, and DWI sequences, however not SWI, where image quality at the level of the posterior fossa receives high scores with both 1Tx32Rx coils, and the various scanning modes.

Sequence-specific analysis demonstrated that PD-weighted imaging derived the greatest benefit from the 8Tx64Rx coil, irrespective of whether pTx was applied. In particular, significant improvements over the 1Tx32Rx coil were observed across all five assessed parameters in both the temporal lobes and posterior fossa. In contrast, SWI demonstrated no significant differences between coils across any assessed parameter in any anatomical region.

The only parameter suggesting potential superiority of the 1Tx32Rx coil was signal homogeneity in DWI within the superior frontal, parietal, and occipital regions. Kruskal–Wallis testing demonstrated a significant overall difference between groups (*p* = 0.02); however, *post-hoc* Dunn's tests with Holm correction did not identify any significant pairwise differences. This suggests that variation was distributed across multiple coil groups, with no individual pairwise comparison reaching statistical significance following correction.

Concerning overall image and diagnostic quality, no significant difference was observed between coils and scan modes, as shown in Table 3, with equivalence between coils and scanning modes. Whilst the average scores for overall image and diagnostic quality were higher for the 8Tx64Rx (CP) coil compared to the 1Tx32Rx coil, and higher for the 8Tx64Rx (pTx) than 8Tx64Rx(CP) coil, there was no statistical difference on Kruskal–Wallis testing (with *p* = 0.13 for diagnostic quality, and *p* = 0.26 for image quality.)

**Table 3:** Maximum SAR (1W) for circularly polarized (CP) excitation for VOPs generated by combined Q-matrices (14 body positions from two body models).

VOP and additional safety factor(s)	Max. 1 W SAR CP mode (W/kg)
Uncompressed Q-matrices from CST export	0.314
Q-matrices + manufacturer amplitude, phase error	0.364
Q-matrices + manufacturer error + simulated/measurement error	0.435
VOP with 25% worst case overestimation + manufacturer error + simulated/measurement error	0.981

**Figure 10:**
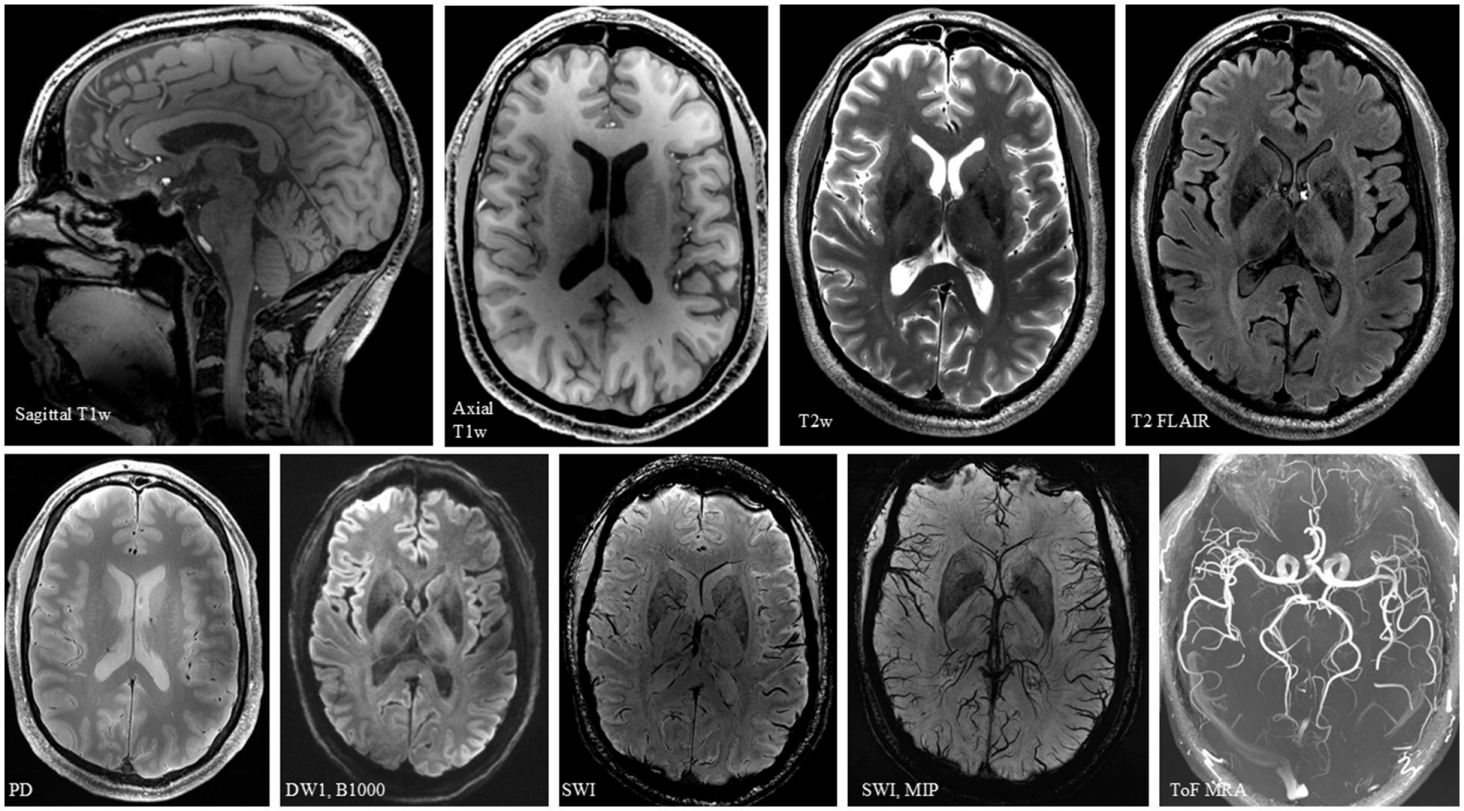
Images of the brain obtained from a subject scanned with the 8Tx64Rx (CP). These include a sagittal T1-weighted acquisition through the midline structures; 3D T_1_-weighted, T_2_ TSE, T_2_ FLAIR, PD, and DWI (*b* = 1,000 s/mm^2^), SWI and SWI Minimum Intensity Projection (MIP) axial acquisitions acquired at a plane through the level of basal ganglia, insula and corona radiata; and TOF MRA (MIP) through the Circle of Willis.

Figure 10 shows examples of imaging (T_1_w, T_2_w, PDw, T_2_ FLAIR, SWI, DWI, and TOF MRA) acquired on scanning a healthy volunteer with 8Tx64Rx (CP). Axial acquisitions are shown at the level of the insula, basal ganglia and corona radiata, together with a sagittal T_1_w midline acquisition. These are representative of the image and diagnostic quality achieved with the 8Tx64Rx (CP).

**Figure 11:**
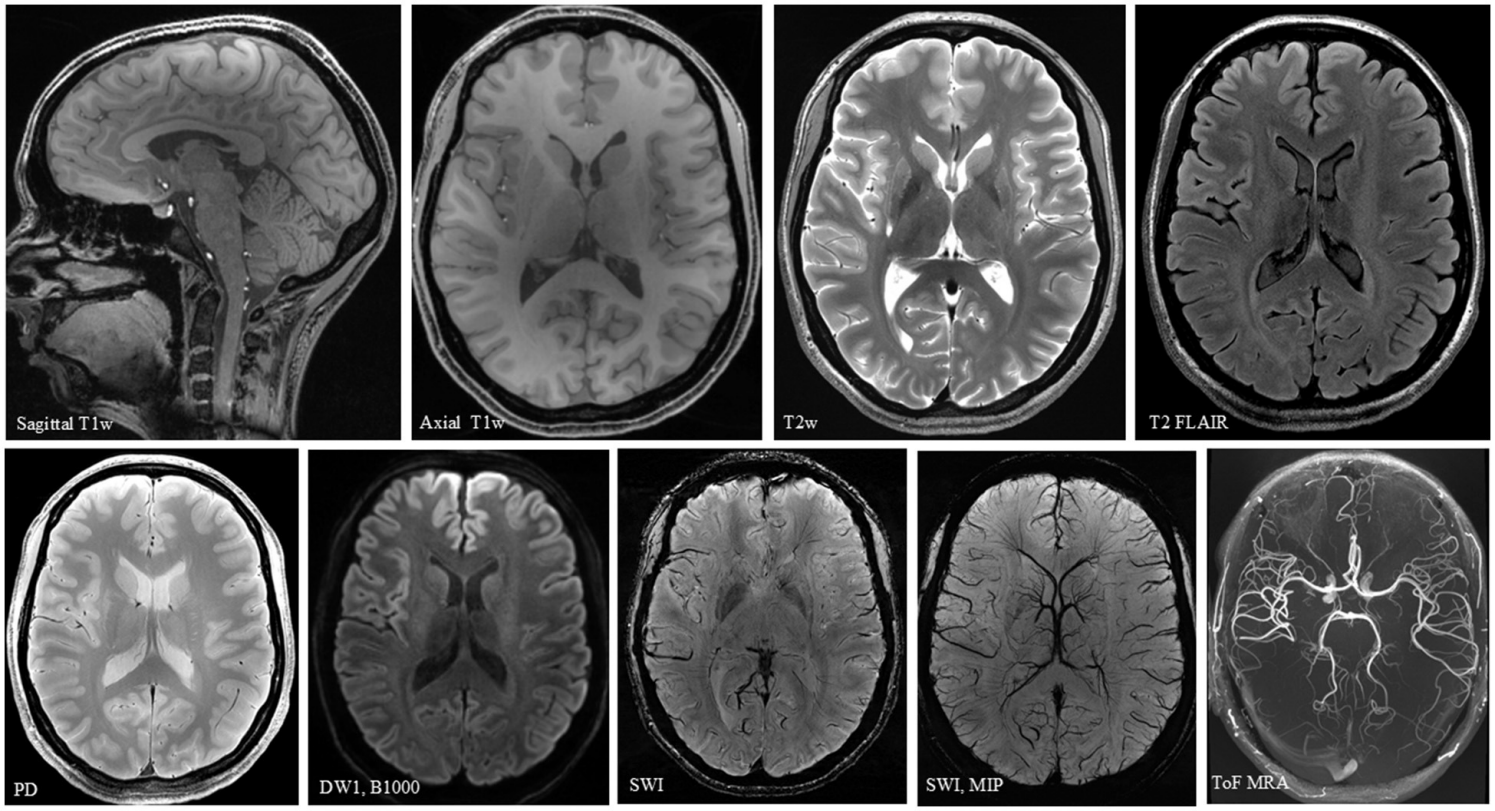
Images of the brain obtained from a subject scanned with the 8Tx64Rx (pTx). These include a sagittal T1-weighted acquisition through the midline structures; 3D T_1_-weighted, T_2_ TSE, T_2_ FLAIR, PD, and DWI (*b* = 1,000 s/mm^2^), SWI and SWI MIP axial acquisitions acquired at a plane through the level of basal ganglia, insula and corona radiata; and TOF MRA (MIP) through the Circle of Willis.

Figure 11 shows examples of imaging (T_1_w, T_2_w, PDw, T_2_ FLAIR, SWI, DWI, and TOF MRA) acquired a different healthy volunteer with the 8Tx64Rx (pTx). Axial acquisitions are shown at the level of the insula, basal ganglia and corona radiata, together with a sagittal T_1_w midline acquisition. These are representative of the image and diagnostic quality achieved with the 8Tx64Rx (pTx).

**Figure 12:**
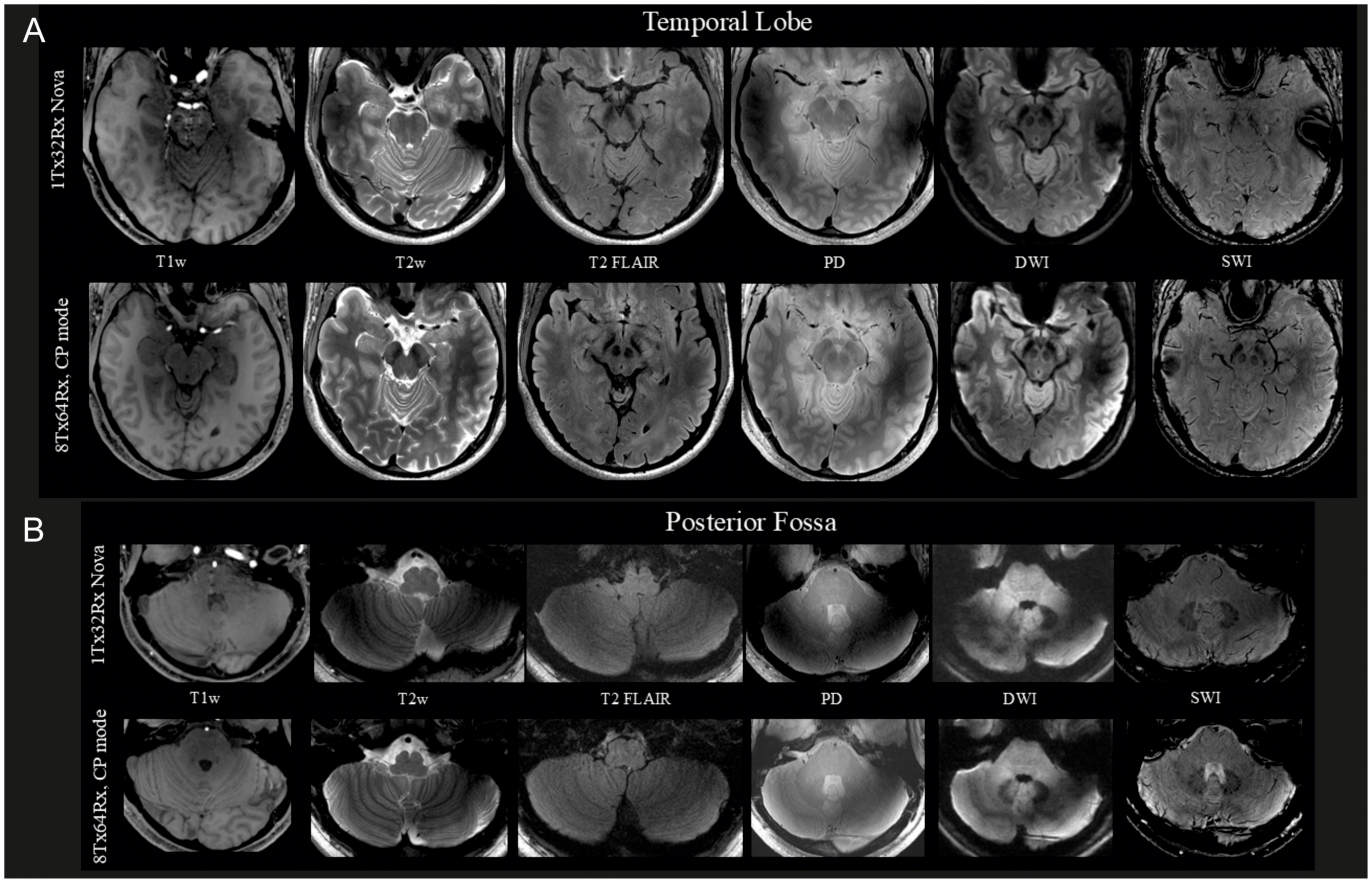
Comparison of images acquired in a healthy volunteer with various sequences (axial 3D T_1_-weighted, T_2_ TSE, T_2_ FLAIR, PD, DWI, and SWI) at the level of the temporal lobes **(A)** and cerebellum/posterior fossa **(B)**, obtained respectively with the 1Tx32Rx coil (top rows) and 8Tx64Rx (CP) (bottom rows).

Figure 12 compares T_1_w, T_2_w, PDw, T_2_ FLAIR, SWI, and DWI sequences at the level of temporal lobes (a) and posterior fossa (b) between the 1Tx32Rx coil (top rows) and 8Tx64Rx (CP) (bottom rows). This show artifacts commonly observed at 7T compared with lower field strength scanners, where inhomogeneity of the B_0_ and ${\text{B}}_{1}^{+}$ fields leads to signal non-uniformity and regional signal loss, particularly at T_2_ FLAIR, DWI and PD sequences. Compared with the 1Tx32Rx coil, 8Tx64Rx (CP) demonstrated improved signal homogeneity, with corresponding improvements in image and diagnostic quality in temporal lobes, cerebellum, and brainstem.

**Figure 13:**
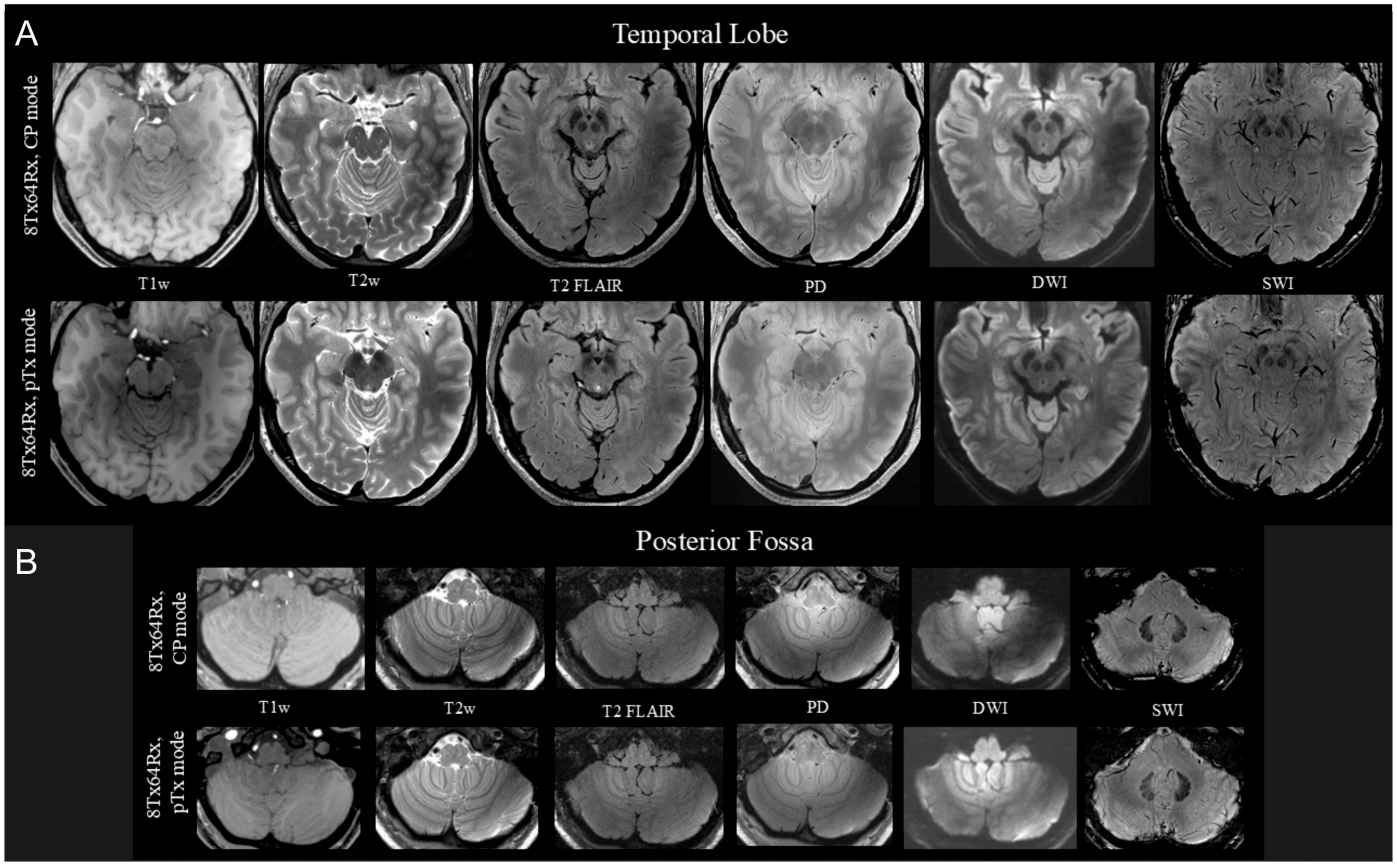
Comparison of images acquired in a different healthy volunteer (compared to Figure 12) with various sequences (axial 3D T_1_-weighted, T_2_ TSE, T_2_ FLAIR, PD, DWI, and SWI) at the level of temporal lobes **(A)** and cerebellum/posterior fossa **(A)**, obtained with the 8Tx64Rx (CP) (top rows) and 8Tx64Rx (pTx) (bottom rows), respectively.

Figure 13 compares T_1_w, T_2_w, PDw, T_2_ FLAIR, SWI, and DWI sequences in a different healthy volunteer at the level of temporal lobes (a) and posterior fossa (b), between the 8Tx64Rx (CP) and 8Tx64Rx (pTx). It illustrates the further improvement in signal homogeneity and, as a result, image and diagnostic quality at the level of the temporal lobe and posterior fossa, which can be achieved scanning with the 8Tx64Rx (pTx) compared to 8Tx64Rx (CP).

#### 3.3.4 Quantitative analysis of image homogeneity

For T2 TSE sequences, the average coefficient of variation between signal in ROIs in different brain regions and hemispheres, was 35.3% ± 8.9% for 1Tx32Rx; 27.7% ± 7.7% for 8Tx64Rx (CP); and 20.2% ± 7.3% for 8Tx64Rx (pTx). A statistically significant improvement was seen for scans acquired with 8Tx64Rx (CP) compared to the 1Tx32Rx coil (*p* = 0.007); with a further statistically significant improvement, when scanning with the 8Tx64Rx (pTx) compared 8Tx64Rx (CP; *p* = 0.008).

For DWI (b = 1000 s/mm^2^) sequences, the average coefficient of variation in different brain regions and hemispheres was 92.6% ± 18% for 1Tx32Rx; 58.7% ± 10% for 8Tx64Rx (CP); and 42.2% ± 17% for 8Tx64Rx (pTx). There was a statistically significant improvement for DWI scans acquired with the 8Tx64Rx (CP) compared to the 1Tx32Rx (*p* = 3.5E−6), and with the 8Tx64Rx (pTx) compared 8Tx64Rx (CP; *p* = 9.5E−4) coil.

For T_2_ FLAIR sequences, the average coefficient of variation between the different was 25.4% ± 10% for 1Tx32Rx; 20.2% ± 11% for 8Tx64Rx (CP); and 17% ± 10% for 8Tx64Rx (pTx). A statistically significant improvement was evident for scans acquired with 8Tx64Rx (CP) compared to the 1Tx32Rx coil (*p* = 0.027); with however no statistically significant improvement, when scanning in 8Tx64Rx (pTx) compared 8Tx64Rx (CP) mode (*p* = 0.082).

Tables 4a,b summarize (a) the coefficients of variations with standard deviations and (b) the statistical significance on using paired Student's *t*-test.

**Table 4:** (a) The coefficients of variations for signal in various brain regions, supra- and infratentorially, and both hemispheres for T2 TSE, T2 FLAIR and DWI (b = 1000 s/mm^2^) sequences for 1Tx32Rx, 8Tx64Rx(CP) and 8Tx64Rx(pTx).

(a) Coefficient of variation	T_2_ TSE	T_2_ FLAIR	DWI (b = 1000 s/mm^2^)
1Tx32Rx	35.3% ± 8.9%	25.4% ± 10%	92.6% ± 18%
8Tx64Rx(CP)	27.7% ± 7.7%	20.2% ± 11%	58.7% ± 10%
8Tx64Rx(pTx)	20.2% ± 7.3%	17% ± 10%	42.2% ± 17%
(b) Paired student *t*-tests	*p*-value		
	T_2_ TSE	T_2_ FLAIR	DWI (b = 1000 s/mm^2^)
1Tx32Rx vs. 8Tx64Rx(CP)	0.007^*^	0.027^*^	3.50E−06^*^
8Tx64Rx(CP) vs. 8Tx64Rx(pTx)	0.008^*^	0.082	9.50E−04^*^

**(b)** The statistical differences for T_2_ TSE, T_2_ FLAIR, and DWI (B1000) between 1Tx32Rx and 8Tx64Rx(CP), and between 8Tx64Rx(CP) and 8Tx64Rx(pTx). Statistical significance between coil configurations is indicated by asterisks (^*^P < 0.05).

### 3.4 SNR and g-factor comparison

**Figure 14:**
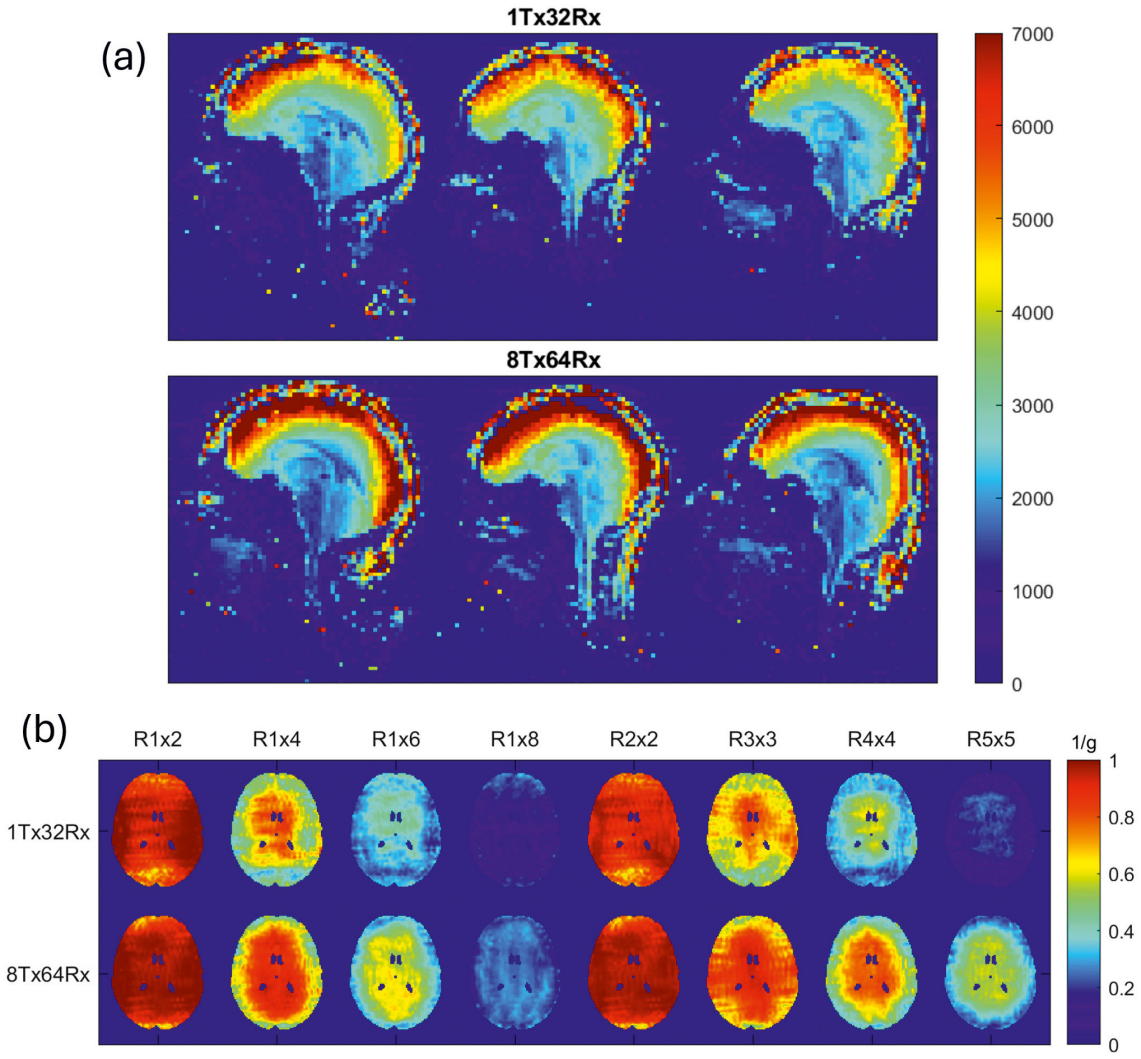
**(a)** Receive SNR maps for all three volunteers comparing the commercially-available 1Tx32Rx coil against the novel 8Tx64Rx coil. **(b)** Simulated 1/g-factor maps averaged across all three volunteers in MNI space for eight different acceleration factors.

As seen from Figure 14a, the receive SNR was 2,057.7 ± 1,298.7 for 1Tx32Rx and 4,087.5 ± 2,376.6 for 8Tx64Rx (*p* = 0.014) in the periphery and 1,613.9 ± 971.8 for 1Tx32Rx, and 1,444.6 ± 844.3 for 8Tx64Rx (*p* = 0.197) in the center. The 1/g-factor maps shown in Figure 14b reflects slight differences between the two coils at low acceleration factors but marked improvements in the 8Tx64Rx coil at high acceleration factors.

## 4 Discussion

### 4.1 Technical considerations

The development of an 8Tx64Rx head coil for 7T imaging comes with technical challenges for coil engineering, validation and practical implementations. High-density receive arrays are inherently more complex to construct than 32-channel arrays, which has historically limited their availability. However, most contemporary 7T platforms are already equipped with 64Rx channels, and the number of installed 7T systems continues to rise globally. Therefore, the present work is not a niche prototype, rather, it reflects a necessary feature for clinical 7T systems as clinicians seek to maximize parallel imaging performance and fully exploit the unique capabilities of UHF scanners.

Validation of a 64-channel receive coil poses additional challenges compared with 32-channel designs. Electromagnetic modeling of high-channel coils becomes substantially more computationally demanding, and including all receive elements in the simulation framework was not feasible within reasonable resource limits. Prior evidence showing a linear and limited effect of receive elements on transmit performance supported the practical decision to exclude the receive array from the electromagnetic model in the present work. Alongside this, coil thermometry validation was complicated by electronic heating of the receive housing arising mainly from the pre-amplifier bias current. In some cases, this heating exceeded the temperature change produced by RF power deposition, thereby masking the RF-induced effect and greatly affecting the accuracy of the MR thermometry results. The implications of this are that more conservative safety factors must be selected for the coil SAR supervision, which potentially limits RF performance. This receive heating challenge is well-recognized in the context of high-element arrays and requires further development to overcome (Williams et al., 2025).

The *in vivo* SNR and g-factor maps results are consistent with theoretical expectation for a 64-channel coil at 7T. Peripheral SNR increased relative to a 32-channel coil, reflecting the higher receive element density, while central SNR remained comparable. It should be noted that SNR evaluation was performed using non-accelerated acquisitions, providing a measure of intrinsic coil performance. In routine scanning, parallel imaging is widely used, and therefore g-factor performance provides a more realistic indicator of achievable imaging performance.

A more practically consequential finding is the improvement in g-factor performance associated with the 64-channel coil. Achieving higher spatial resolution necessitates more phase-encoding steps, resulting in longer acquisition times. The improved g-factor performance of the 64-channel coil enables higher parallel imaging acceleration before central noise amplification and parallel imaging artifacts compromise diagnostic quality. As a result, high-resolution acquisitions can be achieved with more effective scan-time reduction than is possible with a 32-channel coil. Shorter examinations have multiple downstream benefits, including improved patient comfort, reduced sensitivity to motion, and enhanced throughput in a clinical setting. On top of that, acceleration does not solely influence efficiency. By shortening echo trains, high-factor parallel imaging reduces ${\text{T}}_{2}^{*}$-related blurring, which is particularly relevant at 7T due to intrinsically shorter ${\text{T}}_{2}^{*}$ values. Likewise, reducing echo spacing in EPI-based acquisitions lessens geometric distortion, a well-known challenge at ultra-high field. In this context, higher channel count directly translates to improved image quality in addition to temporal efficiency.

It is also important to note that the imaging sequences used in this study were largely derived from sequences originally optimized for a 32-channel coil operating in CP mode. As such, they do not yet represent the full capacity of this hardware and there remains substantial scope for further improvement. For instance, many of the pTx sequences only used volumetric ${\text{B}}_{1}^{+}$ shimming, which is suboptimal compared to dynamic pTx for improving ${\text{B}}_{1}^{+}$ inhomogeneity.

A practical limitation of pTx is the additional workflow overhead associated with subject-specific ${\text{B}}_{1}^{+}$ mapping and pulse calculation, which may impact clinical translation. However, new developments in Universal Pulses (UPs) (Gras et al., 2016) may help reduce the need for subject-specific calibration and thereby mitigate workflow constraints. Following this study, UPs for the 8Tx64Rx could now be generated with the volunteer data collected in this study and then readily applied to a variety of 3D sequences available in the PASTeUR package (Gras et al., 2019). Adapting 2D sequences with pTx is a greater challenge, since each slice-selective pulse requires optimization which can be computationally expensive (instead of a single volumetric pulse for 3D). In this study, a custom-built slice-by-slice shimming approach was successfully used in the RESOLVE DWI scans and has been shown to be robust to inter- and intra-scan repeatability tests (Ding et al., 2024). With appropriate sequence programming adaptations, a similar approach could also be taken for the TSE and FLAIR 2D acquisitions. Although this implementation is an in-house sequence, it is not inherently restricted to the present hardware and could be applied to other pTx-enabled systems. Wider dissemination, for example as a research package (e.g., C2P) within vendor-supported frameworks such as Siemens Healthineers' Teamplay platform, could facilitate broader evaluation and potential clinical adoption. An additional option for improving 2D sequences without a large computational burden could be to implement newer approaches such as MetaPulse2D (Mauconduit et al., 2022) or to use deep learning methods for 2D pTx pulse design. Not only might these advanced dynamic pTx modifications help improve ${\text{B}}_{1}^{+}$ homogeneity, but it is also possible that the additional degrees of freedom can be exploited in pulse design to reduce the local SAR burden for some sequences.

On the receive side, optimization for a 64-channel coil would allow even greater utilization of the available parallel imaging performance such as higher acceleration factors which is likely to provide further gains in both resolution and efficiency. In addition, some vendor-supplied reconstruction filters behaved differently with the altered receive sensitivity profile of the 64-channel coil. Disabling these filters proved adequate for the present study, but dedicated reconstruction pipelines and filtering strategies tailored to high-density arrays would be preferable. Overall, the protocols used here provide a solid basis for comparison, but there is still substantial scope for optimization for high-channel, parallel-transmit enabled coils.

Lastly, from a clinical workflow perspective, the comparison between the 1Tx32Rx and 8Tx64Rx coil in CP mode introduces minimal practical differences, aside from the requirement to operate the system in pTx mode. This may require a one-time mode-switching step (on the order of several minutes) in the beginning of the day on the Terra scanner, but can be maintained across all subjects and is no longer required on the newer Terra.X platform. The use of pTx introduces some additional considerations related to pulse calibration and optimization. However, with current sequence implementations, most acquisitions are executed in a manner largely comparable to conventional CP workflows from a user perspective. While subject-specific pulse design may introduce a short preparatory delay prior to acquisition, approaches such as Universal Pulses can mitigate this, enabling workflow comparable to standard imaging. Universal pulses would decrease operator training times and enable a range of operators to perform clinical scanning, with potential time saving too. Overall, these findings support the clinical feasibility of using 8Tx64Rx coil.

### 4.2 Clinical considerations and neuroimaging validation study

Although this study was conducted in healthy volunteers, the 8Tx64Rx coil was designed with future clinical translational use in mind. Requirements for clinical UHF imaging differ from those in a research environment, with design of our coil tailored to achieve ultra-high resolution neuroimaging, and to address the needs of clinical neuroimaging. High-quality, homogeneous imaging throughout the entire brain for a variety of sequences is a prerequisite for clinical neuroimaging at 7T, particularly given the known challenges of B0 and ${\text{B}}_{1}^{+}$ inhomogeneity at UHF. A clinical working environment relies on a fast, efficient workflow and throughput. Increasing patient comfort and minimizing claustrophobia, leads to decreased patient motion and consequently lesser imaging artifacts. Technical advance, in the form of a 64-channel design, not only improves image quality but can also decrease scan time. Easy communication between patients and operators is essential for safe operating. The mechanical and electrical design of the 8Tx64Rx coil presented focuses on image quality throughout the brain, operator ease and workflow, and participant comfort, making it a promising, versatile tool for clinical and research settings.

The data presented demonstrate the high image quality obtained with the 8Tx64Rx coil in CP and pTx mode. Performance of the 8Tx64Rx coil was equal to or superior across most evaluated metrics, when compared with the commercial 1Tx32Rx coil, which currently represents the regulatory-approved 7T clinical standard, supporting therefore the primary objective of this work. The 8Tx64Rx coil achieved improved signal homogeneity, image and diagnostic quality in areas of concern for UHF imaging, namely temporal lobes and posterior fossa. The most marked improvements were observed in sequences known to be particularly sensitive to B0 and ${\text{B}}_{1}^{+}$ inhomogeneities, including PDw, T_2_ FLAIR and DWI, which rely on high flip-angle refocussing pulses. This was also reflected in the signal coefficients of variations in different brain regions, with in particular a marked statistical difference for DWI, heavily influenced by ${\text{B}}_{1}^{+}$ field inhomogeneities. The observed gains likely reflect improved ${\text{B}}_{1}^{+}$ homogeneity and receive sensitivity, particularly in pTx mode. Signal homogeneity translates to higher diagnostic quality. As anticipated, improvements were smaller for SWI and TOF angiography, where image quality with the 1Tx32Rx coil is already high.

Overall though, signal homogeneity in the posterior fossa and temporal lobes remained inferior to that observed in more cranial brain regions, as expected at 7T. However, the pTx capability of the 8Tx64Rx coil contributed to measurable improvements in these areas. Notably, improvements were already apparent in CP mode compared with the 1Tx32Rx coil, with further enhancement observed under pTx operation. Continued development of more sophisticated pTx strategies will likely be necessary to mitigate residual inhomogeneity further, especially for PD, DWI, and T_2_ FLAIR imaging.

Scores for overall image and diagnostic scan quality, reflected the Radiologists' assessment of the diagnostic potential of a scan. Whilst the average rating for overall image and diagnostic quality was higher for 8Tx64Rx (CP) compared to 1Tx32Rx, and for 8Tx64Rx (pTx) compared to 8Tx64Rx (CP), there was no statistical difference, with relatively high standard deviations noted. There was amongst the Radiologists, a range of experience in 7T MRI and with research radiological scoring (with two Radiologists principal investigators and one investigator, in an equivalent 7T MRI medical device study). The variation in scores may reflect these differences in experience, with scores noted to be frequently lower for those Radiologists with lesser 7T, but extensive clinical 1.5T and 3T experience. Average scores ranging from “acceptable” to “good,” also highlight the discussed shortfalls of 7T MRI related to signal inhomogeneity and increased artifacts. There will be an expected need for clinicians to familiarize themselves with appearances at 7T, when centers are transitioning from clinical 3T to 7T neuroimaging.

Operator experience is also an important practical consideration. Coils at 7T incorporate both transmit and receive elements, and are inherently heavier than receive-only coils at lower field strengths. To mitigate this, the mechanical design was kept as compact as possible to facilitate positioning and handling. This is reflected in the operator scoring, where the 8Tx64Rx coil was rated as significantly easier to handle and move compared with the 1Tx32Rx system.

With respect to protocol handling during scanning, the 1Tx32Rx coil demonstrated a numerically higher mean score, although this difference was not statistically significant. It is important to note that operator scoring was performed by radiography and physics operators with varied backgrounds and expertise, some not familiar with pTx but experienced in using the 1Tx32Rx coil, and others with pTx expertise. This may have influenced subjective assessments of workflow and protocol handling. As the 1Tx32Rx platform represents an established and routinely used system, increased training and experience with pTx workflows would be expected to reduce this difference over time.

There was no difference in “ease of communication” during scanning between coils and scanning modes. We hypothesize, that in a clinical neuro-setting, where good communication is vital, there may be advantages seen scanning with the open-faced 8Tx64Rx coil with its outlook mirrors; for example during fMRI for assessment of eloquent brain areas in surgical tumor and epilepsy patients.

As 7T MRI is gradually being introduced into clinical neuroimaging, consideration of patient experience is increasingly important. The open-face geometry of the 8Tx64Rx coil allows individuals to see out of the coil, more closely resembling conventional receive-only 1.5T and 3T head coil configurations, with the aim of reducing claustrophobia and anxiety while facilitating communication with the operator. Across all scans, participant-reported comfort was high, and claustrophobia scores were low, and as a result, no examinations required exclusion from analysis due to motion artifact. However, no statistically significant differences in tolerability were observed between coils or transmission modes, despite the open-face configuration. The generally high scores given for all scans may reflect in part that healthy volunteers participating in these studies are generally motivated, able to tolerate longer scanning, and are self-selecting with regards to claustrophobia. It is difficult to ascertain, if patients living with neurological conditions would be more discerning in their scores.

Participants were also encouraged to provide any additional comments. It was noted that one lower score for the 8Tx64Rx (pTx) coil related to the participant feeling dizzy on entering the scanner. Two participants had noticeable differences in scoring between CP and pTx modes, including a high claustrophobia score during the pTx acquisition, not reported for the CP mode scan. One of them described difficulty in interpreting the questionnaire wording, particularly as the claustrophobia scale was reversed relative to other items. As there was no objective basis for exclusion, these data were retained in the analysis. Nevertheless, this observation will inform questionnaire design in future studies.

Finally, interpretation of 7T data may involve a learning curve, particularly when novel hardware configurations are introduced. The 8Tx64Rx design incorporates a substantially higher receive channel density, resulting in increased peripheral receive sensitivity, which is also demonstrated in the SNR mapping. While increased signal is generally advantageous, the enhanced cortical signal intensity observed with the 64-channel configuration altered visual contrast in certain sequences. In DWI, and to a lesser extent on T_2_ FLAIR, this manifested as relative cortical brightening in the superior frontal, parietal and occipital regions, contributing to lower homogeneity scores for the 8Tx64Rx coil.

The 8Tx64Rx coil and study design were based on the experience gained at our institution of scanning not only healthy volunteers, but patients with a variety of neurological conditions; including inflammatory conditions, such as multiple sclerosis (MS); neurodegenerative conditions, such as motor neurone disease and movement disorders; chronic neurological conditions such as epilepsy; glial brain tumors; and neurovascular conditions, including small vessel disease, cerebral amyloid angiopathy, vasculopathies, with the 1Tx32Rx coil (Keith et al., 2024; Fullerton et al., 2024; Neilson et al., 2026; Keith et al., 2026). Information gained from patient opinion and questionnaires, radiography and operator experiences, physics assessment and radiology assessments, and clinical neuroradiology, neurology, neurosurgery and neuro-oncology opinions was taken account of for the coil design and the study design. We acknowledge though that a limitation of this study is, that only healthy volunteers were scanned, with healthy volunteers self-selecting and motivated. This may lead to lesser motion artifact and likely contributes to the generally high scores for all different coils and scanning modes, leading to little differentiation of coils by the participants, by the participants, as discussed above. Through assessment of contrast-to-noise ratios, lesion conspicuity, ease of performing advanced techniques, such as spectroscopy, using the 8Tx64Rx coil in patients with neurological conditions is required for advanced validation of the coil.

The absence of patient data as part of this study, limits generalization of conclusions to diagnostic use in patients with neurological conditions, and further assessment of the 8Tx64Rx coil and scanning modalities in a variety of conditions is now required, following the results of this study. Based on this, several further, follow-on clinical studies are currently ongoing in our institution. This includes the use of the 8Tx64Rx coil in patients with MS; in patients with temporal lobe epilepsy making additionally use of advanced imaging techniques such as ^1^H MR spectroscopy (MRS), arterial spin-labeling (ASL), diffusion tensor imaging (DTI) and fMRI; and in patients with glioma with scans including also ^1^H MRS and spectroscopic imaging (MRSI). Once completed, we expect these to provide further information on clinical suitability and performance of the 8Tx64Rx coil in clinical scenarios and highlight any possible issues or difficulties, that may need addressed.

Although this appearance is consistent with the expected receive profile of a high-density array, it may initially be perceived to mimic or mask pathology, especially when compared to 3T imaging, where cortical signal is typically more uniform. It remains to be determined whether increasing interpretative familiarity alone would reduce this perception, or whether further optimization through filtering or advanced reconstruction approaches, such as Uniform Combined Reconstruction (UNICORN) (Chebrolu et al., 2019), could moderate the visual prominence of peripheral signal weighting.

### 4.3 Future work

Having demonstrated performance that is equivalent or superior to the regulatory-approved 1Tx32Rx 7T head coil, future work will focus on advancing the 8Tx64Rx coil toward broader clinical translation. This is particularly timely given recent regulatory approval of a clinical pTx system, and ongoing efforts are directed toward obtaining approval for the present coil design while further developing the associated hardware, software and acquisition strategies required for routine clinical implementation.

From a hardware perspective, although the 64-channel configuration provided clear improvements in peripheral signal sensitivity, gains in central SNR were limited. Central brain regions, including subcortical structures such as the hippocampus, are clinically important, particularly in mesial temporal sclerosis in epilepsy patients. Future refinements of the coil design will therefore explore strategies to enhance central sensitivity while preserving the peripheral homogeneity benefits observed with the current configuration.

The sensitivity enhancement of 64 channels is expected to have benefits in the case of functional MRI by increasing both spatial sensitivity and temporal SNR (tSNR). This has been demonstrated in the same receive-array design built for a NexGen 7T system (Feinberg et al., 2023) and in current ongoing cognitive neuroimaging studies at the University of Glasgow as well as other sites who we have reproduced the 8Tx64Rx coil for. Another study compared tSNR in gradient-echo fMRI scans using the commercially available 1Tx32Rx and 8Tx32Rx coil when both were used in CP mode. Despite the higher efficiency of ${\text{B}}_{1}^{+}$ with the 8Tx32Rx, the results between both coils without using pTx were similar, likely due to relatively small flip angles used (Graf et al., 2026).

A central area of development of UHF imaging revolves around pTx. As such, future work will focus on expanding incorporation of pTx into a broader range of pulse sequences and further refining pulse design strategies for different clinical applications. In addition, simplification of pTx workflow will be important for broader clinical usability. Development of streamlined pTx implementation, including universal pulse approaches and automated optimization pipelines, may reduce operator dependency and facilitate adoption in centers without dedicated pTx expertise.

At the time of writing this manuscript, the Siemens Magnetom Terra.X scanner had received regulatory approval for clinical use with the Nova 8Tx32Rx head coil. Although the receive SNR and parallel imaging performance are expected to be comparable between our reference 1Tx32Rx coil and the 8Tx32Rx coil, performance benchmarking of our 8Tx64Rx head coil with the 8Tx32Rx head coil remains to be established, and such a study would strengthen the translational relevance of this work.

## 5 Conclusion

Neuroimaging at 7T presents distinct technical and practical challenges compared with lower field strengths, particularly with respect to transmit field inhomogeneity and whole-brain signal uniformity. In this work, we demonstrate that a bespoke 8Tx64Rx head coil design, where high receiver count combined with pTx capability, can achieve image quality that is equivalent or superior to the current regulatory-approved 1Tx32Rx clinical standard across the majority of evaluated sequences.

Improvements were most evident in sequences sensitive to ${\text{B}}_{1}^{+}$ inhomogeneity, with enhanced signal homogeneity in anatomically challenging regions such as the temporal lobes and posterior fossa, while maintaining performance in sequences where baseline image quality was already high. The coil additionally demonstrated significantly improved handling characteristics and high participant tolerability within the volunteer cohort.

Together, these findings support the feasibility of high-channel-count, pTx-enabled head coils for translational 7T neuroimaging and provide a foundation for continued optimization toward broader clinical implementation.

## Data Availability

The original contributions presented in the study are included in the article/Supplementary material, further inquiries can be directed to the corresponding author.
