# Supplementary material for: Evaluation of an open-face 8-channel transmit 64-channel receive 7T head coil for neuroimaging
Source: Front Neurosci. 2026 Jun 19;20:1811488. doi: 10.3389/fnins.2026.1811488 (PMC13328354; doi:10.3389/fnins.2026.1811488)
Supplement: Supplementary file 1 [file Data_Sheet_1.docx]

Supplementary Material

**
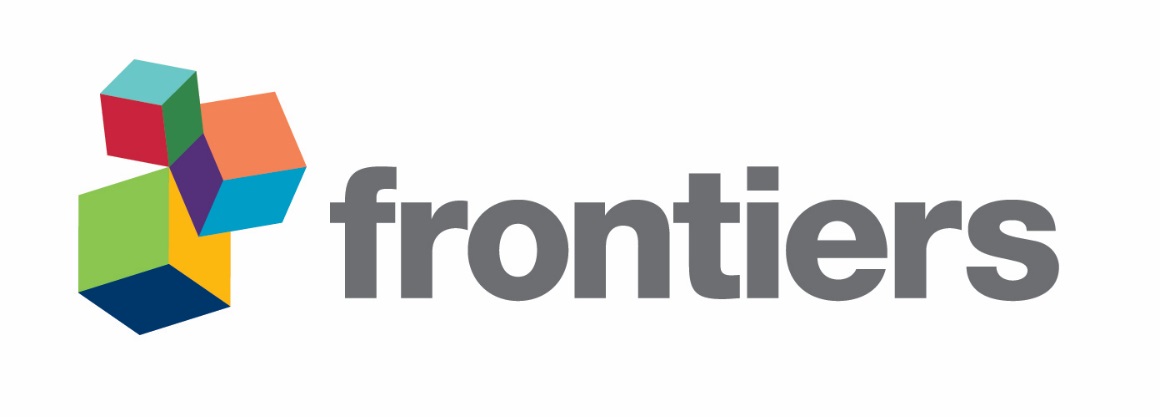
**

**Supplementary Material for**

# “Evaluation of an Open-face 8-channel Transmit 64-channel Receive 7T Head Coil for Neuroimaging”

**Corresponding Author: Shajan Gunamony**

**Supplementary Figure 1. Operator Assessment**

For each coil, the operator (radiographer or physicist) completed the following questionnaire based on a 5-point Likert scale. Each parameter was scored 1 to 5, where 1 is ‘very unhappy’, 2 is ‘unhappy’, 3 is ‘neutral’, 4 is ‘satisfied and 5 is ‘highly satisfied’.

# Operator Assessment

| **1Tx32Rx / 8Tx64Rx (CP) / 8Tx64Rx (pTx)** | | | |
| --- | --- | --- | --- |
| **Parameter Assessed** | **Radiographer score** | **Physicist score** | **Comments** |
| Friendliness of user interface |  |  |  |
| Handling and moving of coil |  |  |  |
| Positioning of coil in scanner |  |  |  |
| Positioning of coil scanned in field-of-view |  |  |  |
| Protocol handling during scanning |  |  |  |
| Ease of communication and / or contact with person scanned |  |  |  |
| Overheating parts (e.g. coils) leading to termination of scans |  |  |  |
| Overall ease of scan |  |  |  |

***Figure S1:*** *Questionnaire used for operator assessment of ease of use of coils during the coil validation study.*

**Supplementary Figure 2.** **Participant Questionnaire**

**Participant Assessment**

| **Questionnaire** | **Strongly disagree** | **Disagree** | **Neutral** | **Agree** | **Strongly agree** |
| --- | --- | --- | --- | --- | --- |
| How comfortable was the scan? |  |  |  |  |  |
| How comfortable was the head coil? |  |  |  |  |  |
| Did you feel claustrophobic? |  |  |  |  |  |
| Did the coil make the contact with the scanning team easy? |  |  |  |  |  |
| If you were a patient, would you be happy to come for this type of examination? |  |  |  |  |  |

| Please rank each scanning session for overall comfort from 1 to 10, with 10 being the best. |
| --- |

| **1^st^ scan** | 1 | 2 | 3 | 4 | 5 | 6 | 7 | 8 | 9 | 10 |
| --- | --- | --- | --- | --- | --- | --- | --- | --- | --- | --- |
| **2^nd^ scan** | 1 | 2 | 3 | 4 | 5 | 6 | 7 | 8 | 9 | 10 |
| **3^rd^ scan** | 1 | 2 | 3 | 4 | 5 | 6 | 7 | 8 | 9 | 10 |

***Figure S2:*** *Questionnaire used for participants during the coil validation study to capture the experience of the healthy volunteers scanned with the different coils and with different scanning modes. The questionnaire used a Likert scale ranging from 1 to 5. In addition, each session was ranked for overall comfort from 1 to 10, with 10 being the best.*

**Supplementary Figure 3.** **Radiology Questionnaire**

**Radiology Assessment**

| **Questionaire** | **1** | **2** | **3** | **4** | **5** |
| --- | --- | --- | --- | --- | --- |
| Overall Image Quality |  |  |  |  |  |
| Overall Diagnostic Quality |  |  |  |  |  |

| **Sequence** | **Questionaire for each sequence** | **1** | **2** | **3** | **4** | **5** |
| --- | --- | --- | --- | --- | --- | --- |
| **3D T1w**  **Superior Frontal / parietal / occipital lobes** | Image Quality |  |  |  |  |  |
|  | Contrast |  |  |  |  |  |
|  | Homogeneity of Signal |  |  |  |  |  |
|  | Artefact |  |  |  |  |  |
|  | Diagnostic Value |  |  |  |  |  |
| **3D T1w**  **Temporal lobes** | Image Quality |  |  |  |  |  |
|  | Contrast |  |  |  |  |  |
|  | Homogeneity of Signal |  |  |  |  |  |
|  | Artefact |  |  |  |  |  |
|  | Diagnostic Value |  |  |  |  |  |
| **3D T1w**  **Posterior Fossa** | Image Quality |  |  |  |  |  |
|  | Contrast |  |  |  |  |  |
|  | Homogeneity of Signal |  |  |  |  |  |
|  | Artefact |  |  |  |  |  |
|  | Diagnostic Value |  |  |  |  |  |

***Figure S3:*** *Sample of the questionnaire used for radiology scoring and diagnostic image quality validation, based on a 5 -point ordinal Likert scale. Scoring was as follows: “1” - “poor”; “2” - “suboptimal”; “3” - “acceptable”; “4” - good; “5” - “excellent”. The overall image and diagnostic quality of the scan was scored, followed by scoring individual sequences and brain regions. Sequences scored included T_1_w, T_2_w, PDw, T_2_ FLAIR, SWI, DWI, and a ToF MRA at the level of the Circle of Willis.*

**Supplementary Figure 4.** **Effect of Receive Array Heating on MR Thermometry Experiment**

**
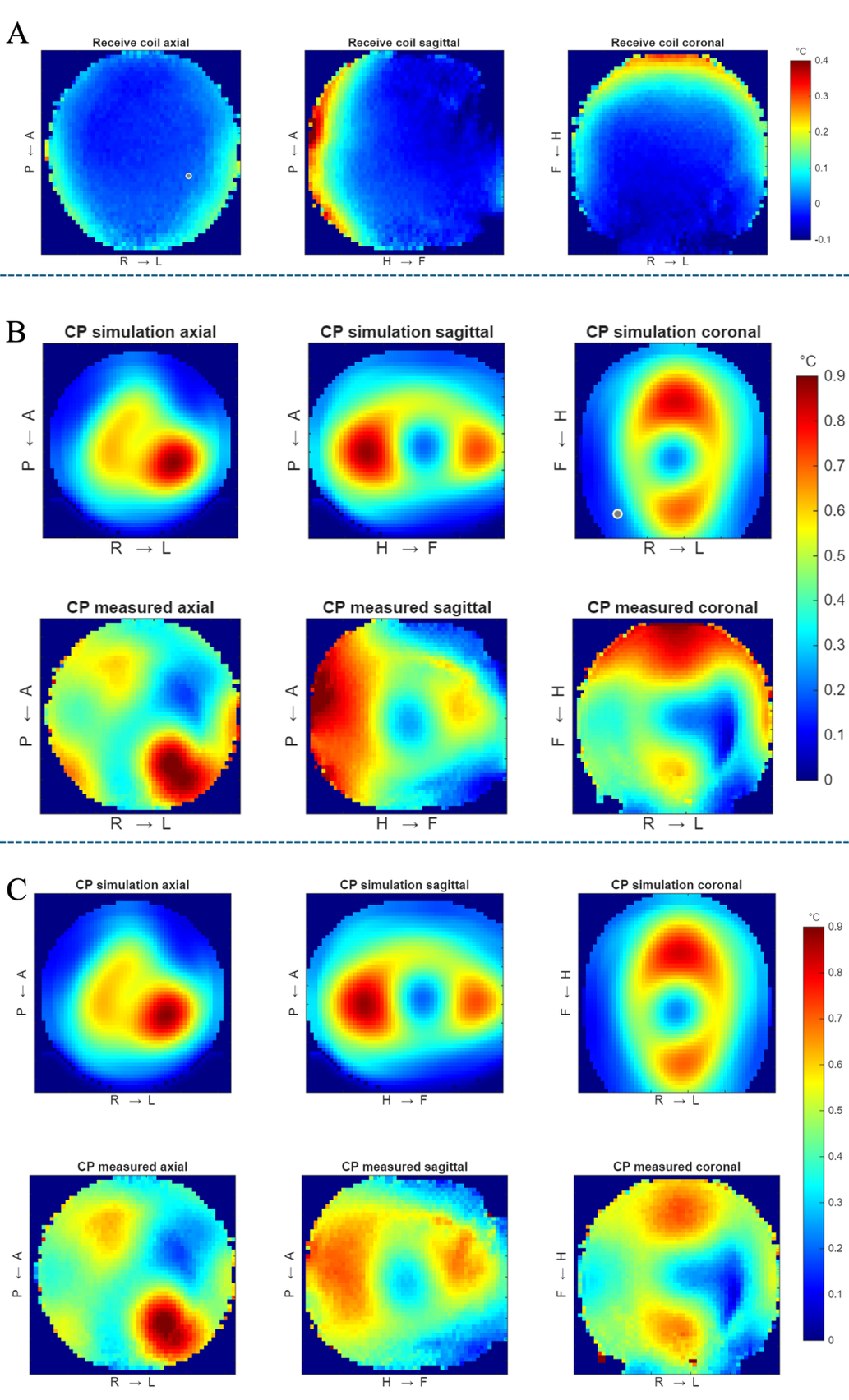
**

***Figure S4:*** *Demonstration of the effect of receive array heating on MR thermometry experiment in CP mode. A) MR thermometry measurement before any RF heating. All temperature changes are attributed to heating of the receive array. B) Comparison of simulation and unprocessed measured temperature maps after RF heating. C) Repetition of Figure 6. Comparison of simulation and RF heating temperature maps after removing the receive array (maps in A subtracted from maps in B).*

**Supplementary Table 1. Statistical analysis of Radiologists’ scores and assessment**

***Table T1:*** *Table showing the statistical analysis of scoring of image quality, diagnostic quality, contrast, signal homogeneity, and artefacts with different sequences and at different brain regions for the 1Tx32Rx coil, and the 8Tx64Rx coil in CP and pTx mode. Scores represent the average score from three radiologists for each score, and differences in scores between coils and transmit modes, which were assessed using the Kruskal-Wallis test. Post hoc pairwise comparison was performed using Dunn’s test with Holm correction for multiple comparisons, where the overall test was significant.*

| **Se-quence** | **Region** | **Assessed parameter** | **1Tx32Rx** | **8Tx64Rx (CP)** | **8Tx64Rx (pTx)** | **p_value** | **Post-hoc testing** | | |
| --- | --- | --- | --- | --- | --- | --- | --- | --- | --- |
|  |  |  |  |  |  |  | **1Tx32Rx vs 8Tx64Rx (CP)** | **1Tx32Rx vs 8Tx64Rx (pTx)** | **8Tx64Rx (CP) vs 8Tx64Rx (pTx)** |
| **All** | Whole brain | Overall diagnostic quality | 3.36 ± 0.84 | 3.59 ± 0.91 | 3.82 ± 0.80 | 0.13 |  |  |  |
|  |  | Overall image quality | 3.44 ± 0.85 | 3.59 ± 0.91 | 3.82 ± 0.73 | 0.26 |  |  |  |
| **DWI** | Posterior fossa | Artefacts | 2.59 ± 1.07 | 2.79 ± 1.00 | 3.08 ± 0.91 | 0.11 |  |  |  |
|  |  | Contrast | 2.90 ± 1.27 | 3.13 ± 1.17 | 3.11 ± 1.06 | 0.75 |  |  |  |
|  |  | Diagnostic value | 1.92 ± 1.02 | 1.87 ± 0.89 | 2.53 ± 0.92 | <0.01 (**) | 0.98 | 0.01 (*) | 0.01 (*) |
|  |  | Image quality | 2.08 ± 1.09 | 2.21 ± 1.03 | 2.76 ± 0.88 | <0.01 (**) | 0.74 | 0.16 | 0.08 |
|  |  | Signal homogeneity | 1.74 ± 0.88 | 1.77 ± 0.81 | 2.45 ± 0.86 | <0.001 (***) | 1.00 | <0.01 (**) | <0.01 (**) |
|  | Superior frontal, parietal, occipital lobes | Artefacts | 4.26 ± 0.59 | 4.03 ± 0.74 | 4.08 ± 0.71 | 0.55 |  |  |  |
|  |  | Contrast | 4.33 ± 0.74 | 4.21 ± 0.86 | 4.24 ± 0.79 | 0.48 |  |  |  |
|  |  | Diagnostic value | 4.05 ± 0.76 | 3.67 ± 1.13 | 3.76 ± 0.97 | 0.33 |  |  |  |
|  |  | Image quality | 4.13 ± 0.73 | 3.74 ± 0.97 | 3.84 ± 0.75 | 0.13 |  |  |  |
|  |  | Signal homogeneity | 3.85 ± 0.81 | 3.26 ± 1.07 | 3.45 ± 0.83 | 0.02 (*) | 0.06 | 0.32 | 0.46 |
|  | Temporal lobes | Artefacts | 3.08 ± 0.90 | 3.31 ± 0.83 | 3.29 ± 0.69 | 0.45 |  |  |  |
|  |  | Contrast | 3.49 ± 1.19 | 3.69 ± 1.10 | 3.76 ± 1.00 | 0.66 |  |  |  |
|  |  | Diagnostic value | 2.31 ± 1.20 | 2.62 ± 1.21 | 2.84 ± 1.03 | 0.16 |  |  |  |
|  |  | Image quality | 2.54 ± 1.17 | 2.85 ± 1.16 | 3.00 ± 1.01 | 0.24 |  |  |  |
|  |  | Signal homogeneity | 2.13 ± 1.00 | 2.36 ± 1.04 | 2.76 ± 0.94 | 0.04 (*) | 1.00 | 0.03 (*) | 0.42 |
| **FLAIR** | Posterior fossa | Artefacts | 2.64 ± 1.01 | 3.00 ± 1.00 | 3.03 ± 0.82 | 0.25 |  |  |  |
|  |  | Contrast | 2.97 ± 1.04 | 3.41 ± 0.99 | 3.29 ± 0.77 | 0.19 |  |  |  |
|  |  | Diagnostic value | 2.67 ± 1.08 | 3.13 ± 1.17 | 3.29 ± 0.87 | 0.05 (*) | 0.23 | 0.04 (*) | 1.00 |
|  |  | Image quality | 2.92 ± 1.16 | 3.33 ± 1.06 | 3.39 ± 0.79 | 0.06 |  |  |  |
|  |  | Signal homogeneity | 2.49 ± 0.97 | 2.97 ± 1.01 | 3.05 ± 0.84 | 0.03 (*) | 0.11 | 0.05 (*) | 1.00 |
|  | Superior frontal, parietal, occipital lobes | Artefacts | 3.69 ± 0.80 | 3.72 ± 0.65 | 3.82 ± 0.73 | 0.66 |  |  |  |
|  |  | Contrast | 3.74 ± 1.09 | 3.69 ± 0.92 | 3.71 ± 0.90 | 0.79 |  |  |  |
|  |  | Diagnostic value | 3.87 ± 1.08 | 3.87 ± 0.92 | 3.89 ± 0.89 | 0.81 |  |  |  |
|  |  | Image quality | 3.92 ± 0.98 | 3.79 ± 0.83 | 3.92 ± 0.78 | 0.60 |  |  |  |
|  |  | Signal homogeneity | 3.92 ± 0.90 | 3.87 ± 0.86 | 3.95 ± 0.73 | 0.66 |  |  |  |
|  | Temporal lobes | Artefacts | 3.00 | 3.05 ± 0.86 | 3.28 ± 0.83 | 3.37 ± 0.75 |  |  |  |
|  |  | Contrast | 3.10 ± 0.82 | 3.41 ± 0.85 | 3.42 ± 0.83 | 0.26 |  |  |  |
|  |  | Diagnostic value | 2.92 ± 0.81 | 3.49 ± 1.05 | 3.61 ± 0.97 | <0.01 (**) | 0.04 (*) | <0.01 (**) | 1.00 |
|  |  | Image quality | 3.08 ± 0.87 | 3.51 ± 0.88 | 3.61 ± 0.75 | 0.02 (*) |  |  |  |
|  |  | Signal homogeneity | 2.82 ± 0.76 | 3.36 ± 0.87 | 3.55 ± 0.83 | <0.001 (***) | 0.01 | <0.001 (***) | 1.00 |
| **PDw** | Posterior fossa | Artefacts | 2.36 ± 0.96 | 2.56 ± 0.82 | 2.97 ± 0.88 | 0.03 (*) | 1.00 | 0.03 (*) | 0.25 |
|  |  | Contrast | 2.33 ± 1.20 | 2.77 ± 1.06 | 3.42 ± 1.11 | <0.01 (**) | 0.59 | <0.001 (***) | 0.06 |
|  |  | Diagnostic value | 1.87 ± 1.00 | 2.13 ± 1.13 | 3.03 ± 1.05 | <0.001 (***) | 1.00 | <0.001 (***) | <0.01 (**) |
|  |  | Image quality | 2.08 ± 1.11 | 2.44 ± 1.02 | 3.18 ± 0.95 | <0.001 (***) | 0.74 | <0.001 (***) | 0.01 (*) |
|  |  | Signal homogeneity | 1.74 ± 0.88 | 2.00 ± 0.95 | 2.92 ± 1.02 | <0.001 (***) | 1.00 | <0.001 (***) | <0.01 (**) |
|  | Superior frontal, parietal, occipital lobes | Artefacts | 4.10 ± 0.85 | 4.13 ± 0.89 | 4.26 ± 0.79 | 0.61 |  |  |  |
|  |  | Contrast | 4.36 ± 0.84 | 4.33 ± 0.70 | 4.45 ± 0.69 | 0.68 |  |  |  |
|  |  | Diagnostic value | 4.03 ± 0.96 | 3.87 ± 1.08 | 4.16 ± 0.95 | 0.70 |  |  |  |
|  |  | Image quality | 4.03 ± 0.87 | 3.87 ± 0.95 | 4.13 ± 0.81 | 0.71 |  |  |  |
|  |  | Signal homogeneity | 3.77 ± 0.93 | 3.51 ± 1.14 | 4.03 ± 0.91 | 0.23 |  |  |  |
|  | Temporal lobes | Artefacts | 2.51 ± 0.94 | 2.95 ± 0.69 | 3.08 ± 0.78 | 0.02 (*) | 0.19 | 0.05 (*) | 0.55 |
|  |  | Contrast | 2.62 ± 0.96 | 3.05 ± 0.86 | 3.29 ± 0.87 | 0.02 (*) | 0.45 | 0.01 (*) | 0.61 |
|  |  | Diagnostic value | 1.82 ± 0.97 | 2.46 ± 1.05 | 2.84 ± 0.97 | <0.001 (***) | 0.04 (*) | <0.001 (***) | 0.40 |
|  |  | Image quality | 2.13 ± 1.08 | 2.74 ± 0.99 | 2.95 ± 0.96 | <0.01 (**) | 0.07 | <0.01 (**) | 1.00 |
|  |  | Signal homogeneity | 1.74 ± 0.88 | 2.26 ± 0.88 | 2.79 ± 0.87 | <0.001 (***) | 0.08 | <0.001 (***) | 0.07 |
| **SWI** | Posterior fossa | Artefacts | 3.82 ± 1.00 | 3.79 ± 1.10 | 3.87 ± 1.04 | 0.99 |  |  |  |
|  |  | Contrast | 4.15 ± 0.96 | 4.21 ± 0.86 | 4.32 ± 0.84 | 0.85 |  |  |  |
|  |  | Diagnostic value | 3.92 ± 1.16 | 3.85 ± 1.14 | 3.97 ± 1.08 | 0.96 |  |  |  |
|  |  | Image quality | 4.00 ± 1.08 | 3.85 ± 1.09 | 4.03 ± 0.94 | 0.85 |  |  |  |
|  |  | Signal homogeneity | 3.67 ± 1.08 | 3.67 ± 1.11 | 3.84 ± 0.97 | 0.91 |  |  |  |
|  | Superior frontal, parietal, occipital lobes | Artefacts | 4.36 ± 0.90 | 4.26 ± 0.75 | 4.47 ± 0.73 | 0.41 |  |  |  |
|  |  | Contrast | 4.54 ± 0.72 | 4.59 ± 0.55 | 4.53 ± 0.65 | 0.69 |  |  |  |
|  |  | Diagnostic value | 4.44 ± 0.85 | 4.44 ± 0.64 | 4.53 ± 0.65 | 0.69 |  |  |  |
|  |  | Image quality | 4.36 ± 0.87 | 4.41 ± 0.72 | 4.45 ± 0.72 | 0.86 |  |  |  |
|  |  | Signal homogeneity | 4.28 ± 0.94 | 4.21 ± 0.86 | 4.39 ± 0.86 | 0.56 |  |  |  |
|  | Temporal lobes | Artefacts | 3.69 ± 0.95 | 3.56 ± 0.91 | 3.63 ± 0.82 | 0.82 |  |  |  |
|  |  | Contrast | 4.15 ± 0.87 | 4.21 ± 0.86 | 4.13 ± 0.88 | 0.95 |  |  |  |
|  |  | Diagnostic value | 3.85 ± 0.99 | 3.85 ± 1.01 | 3.76 ± 1.02 | 0.97 |  |  |  |
|  |  | Image quality | 3.85 ± 0.99 | 3.87 ± 0.95 | 3.87 ± 0.96 | 1.00 |  |  |  |
|  |  | Signal homogeneity | 3.64 ± 0.84 | 3.59 ± 0.88 | 3.53 ± 0.83 | 0.82 |  |  |  |
| **T1w** | Posterior fossa | Artefacts | 2.72 ± 0.94 | 3.38 ± 1.07 | 3.45 ± 0.95 | <0.01 (**) | 0.02 (*) | <0.01 (**) | 1.00 |
|  |  | Contrast | 4.00 | 3.38 ± 0.85 | 4.03 ± 1.01 | 4.21 ± 0.84 |  |  |  |
|  |  | Diagnostic value | 3.00 | 2.87 ± 1.10 | 3.74 ± 1.09 | 3.95 ± 0.98 |  |  |  |
|  |  | Image quality | 3.05 ± 0.86 | 3.82 ± 0.94 | 4.00 ± 0.99 | <0.001 (***) | <0.01 (**) | <0.001 (***) | 1.00 |
|  |  | Signal homogeneity | 3.10 ± 1.07 | 3.77 ± 1.18 | 3.87 ± 0.93 | 0.01 (*) | 0.03 (*) | 0.02 (*) | 1.00 |
|  | Superior frontal, parietal, occipital lobes | Artefacts | 3.69 ± 0.69 | 4.18 ± 0.68 | 4.16 ± 0.68 | <0.01 (**) | 0.02 (*) | 0.02 (*) | 0.97 |
|  |  | Contrast | 4.49 ± 0.60 | 4.67 ± 0.53 | 4.76 ± 0.49 | 0.11 |  |  |  |
|  |  | Diagnostic value | 4.28 ± 0.65 | 4.64 ± 0.58 | 4.61 ± 0.55 | 0.03 (*) | 0.04 (*) | 0.11 | 1.00 |
|  |  | Image quality | 4.21 ± 0.66 | 4.54 ± 0.60 | 4.61 ± 0.55 | 0.02 (*) | 0.09 | 0.03 (*) | 1.00 |
|  |  | Signal homogeneity | 4.36 ± 0.78 | 4.62 ± 0.75 | 4.68 ± 0.57 | 0.11 |  |  |  |
|  | Temporal lobes | Artefacts | 3.77 ± 0.74 | 3.97 ± 0.78 | 4.00 ± 0.84 | 0.54 |  |  |  |
|  |  | Contrast | 4.18 ± 0.72 | 4.51 ± 0.68 | 4.45 ± 0.72 | 0.12 |  |  |  |
|  |  | Diagnostic value | 4.00 ± 0.79 | 4.36 ± 0.87 | 4.32 ± 0.81 | 0.14 |  |  |  |
|  |  | Image quality | 4.03 ± 0.74 | 4.36 ± 0.81 | 4.34 ± 0.78 | 0.13 |  |  |  |
|  |  | Signal homogeneity | 4.05 ± 0.79 | 4.23 ± 0.90 | 4.26 ± 0.79 | 0.55 |  |  |  |
| **T2w** | Posterior fossa | Artefacts | 3.46 ± 1.00 | 3.97 ± 0.93 | 4.11 ± 0.65 | <0.01 (**) | 0.05 | 0.01 (*) | 0.89 |
|  |  | Contrast | 3.90 ± 0.85 | 4.18 ± 1.00 | 4.45 ± 0.76 | 0.03 (*) | 0.33 | 0.02 (*) | 1.00 |
|  |  | Diagnostic value | 3.00 ± 1.26 | 3.69 ± 1.32 | 4.03 ± 1.17 | <0.01 (**) | 0.07 | <0.01 (**) | 0.60 |
|  |  | Image quality | 3.26 ± 1.21 | 3.74 ± 1.23 | 4.11 ± 1.16 | <0.01 (**) | 0.27 | <0.01 (**) | 0.38 |
|  |  | Signal homogeneity | 2.85 ± 1.16 | 3.44 ± 1.27 | 3.74 ± 1.03 | <0.01 (**) | 0.06 | <0.01 (**) | 0.68 |
|  | Superior frontal, parietal, occipital lobes | Artefacts | 4.67 ± 0.62 | 4.28 ± 0.94 | 4.53 ± 0.83 | 0.06 |  |  |  |
|  |  | Contrast | 4.51 ± 0.72 | 4.31 ± 0.89 | 4.50 ± 0.76 | 0.28 |  |  |  |
|  |  | Diagnostic value | 4.51 ± 0.82 | 4.15 ± 0.93 | 4.37 ± 0.94 | 0.06 |  |  |  |
|  |  | Image quality | 4.54 ± 0.76 | 4.15 ± 0.78 | 4.37 ± 0.91 | 0.03 (*) | 0.11 | 0.45 | 0.33 |
|  |  | Signal homogeneity | 4.51 ± 0.82 | 4.18 ± 0.97 | 4.50 ± 0.76 | 0.24 |  |  |  |
|  | Temporal lobes | Artefacts | 3.44 ± 0.72 | 3.85 ± 0.84 | 3.87 ± 0.74 | 0.05 |  |  |  |
|  |  | Contrast | 3.79 ± 0.77 | 4.10 ± 0.82 | 4.16 ± 0.79 | 0.08 |  |  |  |
|  |  | Diagnostic value | 3.15 ± 1.06 | 3.77 ± 1.09 | 3.84 ± 0.95 | <0.01 (**) | 0.05 (*) | 0.02 (*) | 0.75 |
|  |  | Image quality | 3.23 ± 0.90 | 3.85 ± 0.93 | 3.82 ± 0.90 | <0.01 (**) | 0.02 (*) | 0.02 (*) | 1.00 |
|  |  | Signal homogeneity | 3.03 ± 0.90 | 3.54 ± 1.05 | 3.61 ± 0.82 | 0.02 (*) | 0.13 | 0.04 (*) | 0.64 |
| **ToF** | Whole brain | Artefacts | 4.41 ± 0.88 | 4.49 ± 0.79 | 4.55 ± 0.65 | 0.27 |  |  |  |
|  |  | Contrast | 4.56 ± 0.68 | 4.62 ± 0.63 | 4.68 ± 0.57 | 0.22 |  |  |  |
|  |  | Diagnostic value | 4.46 ± 0.82 | 4.33 ± 0.84 | 4.61 ± 0.64 | 0.12 |  |  |  |
|  |  | Image quality | 4.33 ± 0.77 | 4.23 ± 0.81 | 4.42 ± 0.64 | 0.23 |  |  |  |
|  |  | Signal homogeneity | 4.56 ± 0.79 | 4.56 ± 0.75 | 4.61 ± 0.72 | 0.32 |  |  |  |
